# A Genome-Wide Association Study Reveals a *BDNF*-Centered Molecular Network Associated with Alcohol Dependence and Related Clinical Measures

**DOI:** 10.3390/biomedicines10123007

**Published:** 2022-11-22

**Authors:** Anastasia Levchenko, Sergey Malov, Alexey Antonik, Anastasia Protsvetkina, Kseniya V. Rybakova, Alexander Kanapin, Alexey N. Yakovlev, Anna Y. Nenasteva, Anton E. Nikolishin, Nikolay Cherkasov, Natalia A. Chuprova, Anna S. Blagonravova, Angelica V. Sergeeva, Tatyana V. Zhilyaeva, Maria K. Denisenko, Raul R. Gainetdinov, Alexander O. Kibitov, Evgeny M. Krupitsky

**Affiliations:** 1 Institute of Translational Biomedicine, Saint Petersburg State University, Saint Petersburg 199034, Russia; 2 Institute of Computer Science and Technologies, Peter the Great Saint Petersburg Polytechnic University, Saint Petersburg 195251, Russia; 3 Department of Algorithmic Mathematics, Saint Petersburg Electrotechnical University, Saint Petersburg 197022, Russia; 4 Department of Geometry, Faculty of Mathematics and Mechanics, Saint Petersburg State University, Saint Petersburg 199034, Russia; 5 Department of Addictions, V.M. Bekhterev National Medical Research Center for Psychiatry and Neurology, Saint Petersburg 192019, Russia; 6 Center for Computational Biology, Peter the Great Saint Petersburg Polytechnic University, Saint Petersburg 195251, Russia; 7 Lipetsk Regional Addiction Hospital, Lipetsk 398006, Russia; 8 Moscow Research and Practical Center for Narcology of the Department of Public Health, Moscow 109390, Russia; 9 Laboratory of Molecular Genetics, Serbsky National Medical Research Center on Psychiatry and Addictions, Moscow 119034, Russia; 10 Human Population Genetics Laboratory, Research Center for Medical Genetics, Moscow 115522, Russia; 11 Privolzhsky Research Medical University, Nizhny Novgorod 603005, Russia; 12 Saint Petersburg University Hospital, Saint Petersburg State University, Saint Petersburg 199034, Russia; 13 V.M. Bekhterev National Medical Research Center for Psychiatry and Neurology, Saint Petersburg 192019, Russia; 14 Laboratory of Clinical Psychopharmacology of Addictions, Saint Petersburg First Pavlov State Medical University, Saint Petersburg 197022, Russia

**Keywords:** alcohol dependence, *BDNF*, comorbidity, gene network, genome-wide association study, sex differences

## Abstract

At least 50% of factors predisposing to alcohol dependence (AD) are genetic and women affected with this disorder present with more psychiatric comorbidities, probably indicating different genetic factors involved. We aimed to run a genome-wide association study (GWAS) followed by a bioinformatic functional annotation of associated genomic regions in patients with AD and eight related clinical measures. A genome-wide significant association of rs220677 with AD (*p*-value = 1.33 × 10^−8^ calculated with the Yates-corrected χ^2^ test under the assumption of dominant inheritance) was discovered in female patients. Associations of AD and related clinical measures with seven other single nucleotide polymorphisms listed in previous GWASs of psychiatric and addiction traits were differently replicated in male and female patients. The bioinformatic analysis showed that regulatory elements in the eight associated linkage disequilibrium blocks define the expression of 80 protein-coding genes. Nearly 68% of these and of 120 previously published coding genes associated with alcohol phenotypes directly interact in a single network, where *BDNF* is the most significant hub gene. This study indicates that several genes behind the pathogenesis of AD are different in male and female patients, but implicated molecular mechanisms are functionally connected. The study also reveals a central role of *BDNF* in the pathogenesis of AD.

## 1. Introduction

Alcohol dependence (AD), according to the *International Statistical Classification of Diseases and Related Health Problems 10th Revision (ICD-10)*, is a cluster of behavioral, cognitive, and physiological phenomena that develop after repeated alcohol use and that include a strong desire to consume alcohol, difficulties in controlling its use, persisting in its use despite harmful consequences, a higher priority given to this consumption than to other activities and obligations, increased tolerance, and a physical withdrawal state. Neurobiological findings implicate opioid, dopaminergic, glutamatergic, and GABAergic neurotransmitter systems in the pathogenesis of AD [1].

The worldwide incidence of AD in 2016 was 8.6% among men and 1.7% among women [2]. The average incidence of AD in Russia in 2016 was 16.5% among men and 3.3% among women (https://www.who.int/substance_abuse/publications/global_alcohol_report/profiles/rus.pdf, accessed on 25 September 2021), but it varies widely depending on the region [3]. For example, the rate of heavy drinking (as a proxy for AD) in 1996 in the traditionally Muslim North Caucasus and Volga regions was approximately one-fifth of that seen in the Ural region [3]. There has been a reduction in alcohol-related harms in Russia, following changes in government alcohol policy measures that started in 2000 [4], although the current incidence of AD in Russia twice the worldwide incidence indicates that more preventive measures are needed.

Nearly 50% of factors predisposing to AD are genetic [5], although this figure may be an underestimation [2]. Numerous genome-wide association studies (GWASs) reported hundreds of genes associated with AD and related clinical phenotypes such as alcohol consumption and problematic drinking [6,7,8,9,10,11,12]. Genetic factors predisposing to AD and other problematic patterns of alcohol consumption are also shared with a number of psychiatric traits, primarily major depression, depressive symptoms, attention deficit hyperactivity disorder, schizophrenia, and bipolar disorder [7,13,14,15,16,17]. Genes [18] and gene networks [19] discovered in GWASs await further confirmation in silico, in vitro, and in vivo in terms of their implication in the pathogenesis of AD. The most robust genetic findings are reported for functional variants in ethanol metabolizing genes coding for alcohol dehydrogenase (ADH) and aldehyde dehydrogenase (ALDH) [7,9,10,14,17,20,21,22,23]. Another replicated functional genetic variant rs6265, which is associated with AD-related phenotypes, is found in the gene coding for brain-derived neurotrophic factor (BDNF) [24,25,26,27].

A GWAS of AD in a population from Russia had not been available in the peer-reviewed scientific literature prior to the present investigation, and reports had been limited only to candidate-gene association studies (for example: [28]). In the present investigation, we ran a GWAS followed by a bioinformatic functional annotation of the associated genomic regions in a cohort of male and female patients with AD and eight additional clinical measures (such as anxiety symptoms, alcohol craving, and amount of alcohol consumed per day). This is the first GWAS of AD performed in Russia. Results of this study suggest that molecular pathways implicated in the pathogenesis of AD are the same in both sexes, but different genes play more prominent roles in men and women. The discovered gene network may contain clues to the understanding of the pathogenesis and to the discovery of new treatment options of AD.

## 2. Materials and Methods

### 2.1. Statistical Power Estimation

**Figure 1:**
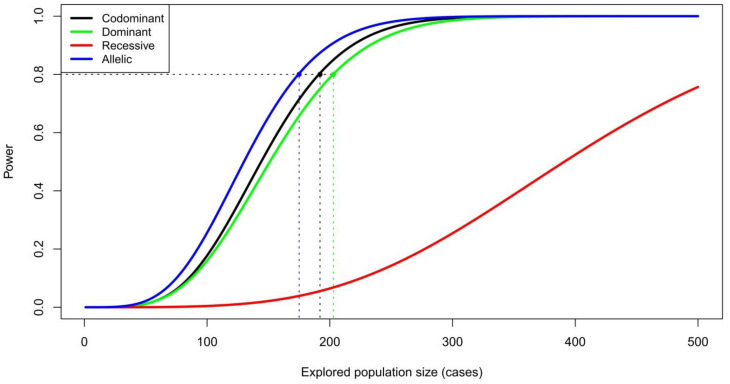
Power of GWAS χ^2^ tests. The necessary number of cases was estimated with the following parameters: the control to case ratio = 5, α = 0.05 (Bonferroni-corrected *p*-value = 1 × 10^−7^, assuming there will 400,000 markers used in the analysis), disease prevalence = 0.1, minor allele frequency in the control group = 0.1, and the allelic odds ratio = 2.5.

We estimated the necessary number of cases given that the control-to-case ratio will be 5. For a χ^2^ test at α = 0.05 (Bonferroni-corrected *p*-value = 1 × 10^−7^, assuming there will be 400,000 markers used in the analysis), disease prevalence = 0.1, minor allele frequency (MAF) in the control group = 0.1, and the allelic odds ratio = 2.5 (the allelic relative risk = 2.05) under Hardy–Weinberg equilibrium (HWE) in both case and control groups, we obtain the number of cases of 192 needed to achieve statistical power of 80% (Figure 1). The statistical power estimation should be used with reservation, because it necessitates prior knowledge of modes of inheritance and of odds ratios and frequencies of alleles associated with the disease. These parameters are unavailable for all genetic variants associated with AD.

### 2.2. Participants

The authors assert that all procedures contributing to this work comply with the ethical standards of the relevant national and institutional committees on human experimentation and with the Helsinki Declaration of 1975, as revised in 2008. All procedures involving human subjects were approved by the Independent Ethics Committee of the V.M. Bekhterev National Medical Research Center for Psychiatry and Neurology (protocol code: EC-2032, excerpt: EC-I-130/20, date of approval: 30 November 2020). Written informed consent was obtained from all subjects.

The study included 224 cases diagnosed with AD according to ICD-10 criteria (F10.2) and ascertained as inpatients in addiction departments of psychiatric hospitals. The patients were also assessed with the following screening instruments (Table 1A–C): (1) A structured clinical interview to assess the self-reported average amount of alcohol consumed and psychiatric and AD history of all first-degree relatives; in addition, the patients indicated the average number of cigarettes smoked daily within the last 90 days, given that tobacco use or nicotine dependence and alcohol dependence are highly comorbid in the Russian population [29]. (2) Alcohol Timeline Followback (TLFB) assessing alcohol consumption and the number of heavy drinking, drinking, and sobriety days within the last 90 days. (3) Ten days after detoxification, patients filled out the Spielberger State-Trait Anxiety Inventory (STAI), Obsessive–Compulsive Drinking Scale (OCDS), Penn alcohol craving scale (PACS), and Visual analogue scale (VAS) for alcohol craving; in addition, answers on the Hamilton Anxiety Scale (HAS) and Montgomery–Asberg Depression Rating Scale (MADRS) were rated by a physician. (4) Finally, patients were assessed with the Clinical Global Impression Scale (CGI)—Severity. Before QC procedures, there were 192 males and 32 females in this cohort, indicating a male/female ratio of 6. The mean age at sampling was 42.2 ± 8.5. Patients reside in Saint Petersburg, Moscow, and Lipetsk, cities in European Russia. The control group included 1059 participants drawn as community volunteers from Moscow, Lipetsk, and Nizhny Novgorod, a city also located in European Russia. There were 860 males and 199 females in this group and their mean age was 39.3 ± 8.6. Individuals reporting any lifetime symptoms indicative of a substance use or psychiatric disorder were excluded as control participants. Cases and controls self-identified as ethnic Russians.

### 2.3. Genotyping and QC Procedures

Extraction of DNA from whole venous blood was performed using the QIAsymphony SP System and the QIASymphonyDNA Midi Kit (QIAGEN, Hilden, Germany). DNA concentration was measured using the Quantus fluorometer (Promega Corporation, Madison, WI, USA). The iScan System and the Infinium Global Screening Array-24 (GSA) v1.0 BeadChip (Illumina, San Diego, CA, USA) were used to genotype 642,824 variants that include markers previously associated with several clinically relevant phenotypes. Preparation of DNA samples was done at the Biobank Center of the Research Park, Saint Petersburg State University, Saint Petersburg, Russia, and genotyping was done at the Federal Research and Clinical Center of Physical-Chemical Medicine, Federal Medical Biological Agency, Moscow, Russia.

Sequential QC procedures were carried out according to recommendations for GWAS data preparation in psychiatric research [30]. Because in some statistical tests patients were compared to controls, whereas in other tests different groups of patients were compared among them, two similar QC procedures were applied (Table 2A,B). Autosomal biallelic single nucleotide polymorphisms (SNPs) having a call rate of at least 98% and individuals with the missing genotyping rate per individual of at most 2% filtered by sex discrepancy passed the primary QC. The cutoffs for minor allele frequency were 1%, whereas for the HWE test the cutoff *p*-values were 10^−6^ for the group of cases and controls and 10^−10^ for the group of cases only. The removed variants also contained SNPs excluded by Illumina in GSA support files (https://emea.support.illumina.com/downloads/infinium-global-screening-array-v1-0-support-files.html, accessed on 1 March 2021) and by dbSNP (https://www.ncbi.nlm.nih.gov/snp/, accessed on 1 March 2021). First- and second-degree relatives as well as potential duplicate samples were determined by calculating identity by descent of all sample pairs [31]. The analysis revealed 57 samples in 30 pairs with the pi-hat value above the threshold of 0.2 (duplicates, first- and second-degree relatives). Following the recommendations in [31], in a pair of related samples we removed the one with the lower call rate (29 samples removed in total).

**Figure 2:**
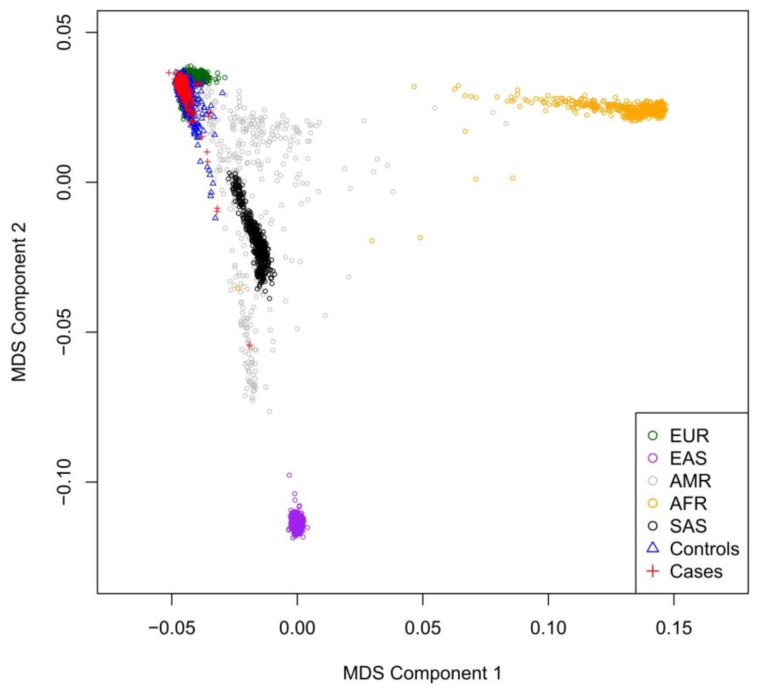
Results of multidimensional scaling of this cohort and several large human populations. Indicated ancestries are: EUR—European, EAS—East Asian, AMR—American, AFR—African, SAS—South Asian. One outlier among cases (red cross) was excluded from the downstream analysis.

**Figure 3:**
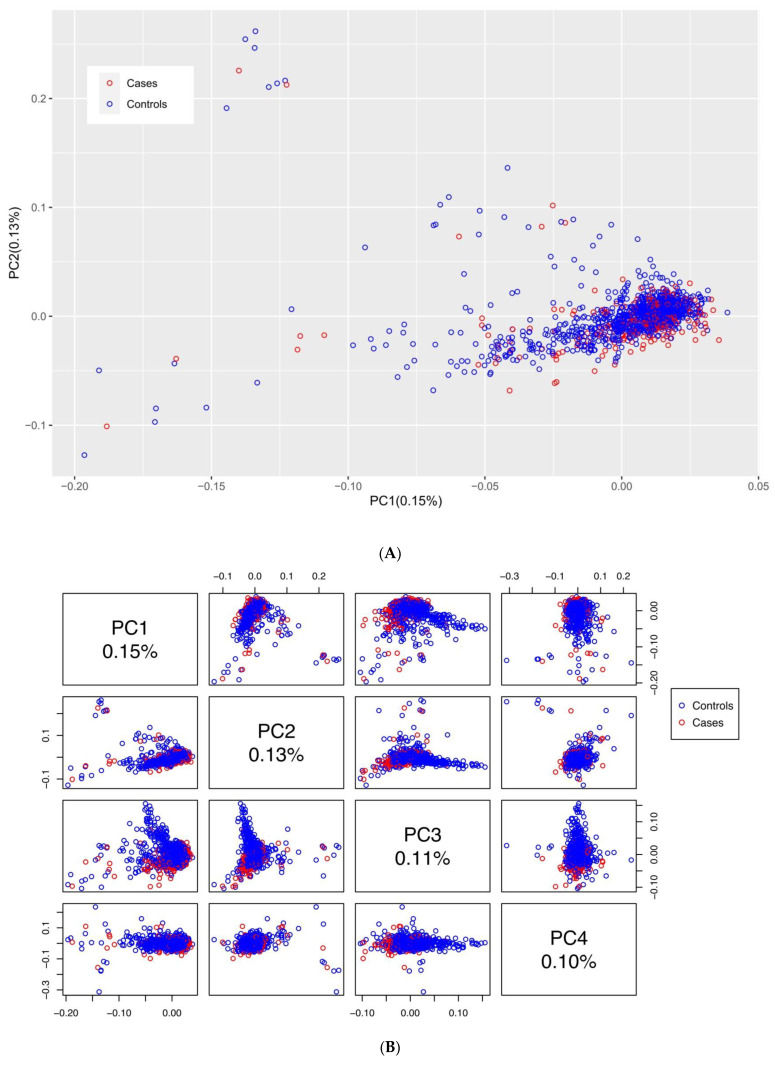
The population genetic structure of cases and controls estimated using the principal component analysis (PCA). The percentages of variance explained by the principal components (PC) are indicated for: (**A**) the first two PC; (**B**) the first four PC.

Next, we harnessed the multidimensional scaling (MDS) approach [30] to place this cohort in the context of several large human populations that represent the world’s genetic diversity. To this end we used the data from the Phase 3 analysis of the 1000 Genomes project (https://genome.ucsc.edu/cgi-bin/hgTables?db=hg38&hgta_group=varRep&hgta_track=tgpPhase3&hgta_table=tgpPhase3&hgta_doSchema=describe+table+schema/, accessed on 1 May 2021) [32]. See Figure 2 that depicts the single outlier among cases; this clear outlier was removed during QC procedures. A principal component analysis (PCA) [33] (after the outlier removal) indicated that cases and controls belong to the same relatively genetically homogenous population (Figure 3A), as the percentage of variance explained by four principal components is small (Figure 3B). The PCA was performed by using the R-package SNPRelate [34] with the preliminary LD-pruning under the standard threshold of 0.2.

In the case–control cohort, 411,586 variants and 1208 individuals passed QC. In the cohort of only patients, 381,084 variants and 192 individuals passed QC (Table 2A,B). The genotyping rate in the resulting datasets was nearly 99.8%. The human genome assembly used throughout this study was GRCh38/hg38.

### 2.4. Association Studies

We decided not to use results of imputation in calculations of genome-wide associations out of concern that the correction for multiple testing will not allow us to make new discoveries. Furthermore, whole genome data from ethnic Russian populations in the same geographic regions than cases and controls are currently insufficient to impute genotypes. Despite this, we attempted to use the data from the 1000 Genomes database, Phase 3, to perform the imputation-based genotype refinement of a candidate region on chromosome 6 and of two alcohol-metabolizing gene regions (see Section 3.1), using Beagle 5.2 [35] with default parameters.

The main GWAS screened for associations of AD in the mixed, male, and female cohorts with any fixed genetic marker (using the genotyped SNPs only) under codominant, dominant, and recessive alternatives, as well as the allelic test (Table S1A). The χ^2^ test was used for the genome-wide screening of associations, and selected genome-wide significant associations were tested more carefully by using the most appropriate statistical test for the contingency table. The odds ratio for 2 × 2 contingency tables or the $\left(1-\rho \right)/\left(1+\rho \right)$ transformation of Pearson’s correlation coefficient (designated as ez2-transformation for the square of the exponentiated Fisher’s z-transformation) for the tables of other sizes define the direction and strength of the association. We used Fisher’s exact test for selected genome-wide significant association tables containing relatively small counts. In addition, logOR Z-test was used if at least one of evaluated marginal probabilities was extremely small. A Bonferroni correction for multiple testing was applied in tests of AD (the main diagnosis), indicating the genome-wide significance (GWS) level of 4.049 × 10^−8^ (411,586 markers tested at α = 0.05 for three groups: the entire cohort, males, and females). The R statistical software (http://www.r-project.org/, accessed on 1 June 2021) [36] was used to complete these analyses.

In the next step, we performed a genome-wide screening for associations of the AD phenotype and regions in the genome by using Fisher’s combined test statistics integrated within the signal localization approach [37]. We used sliding localization windows of sizes 21 (radius = 10), 41 (radius = 20), 71 (radius = 35), 101 (radius = 50), and 201 (radius = 50) SNPs, as well as 100 thousand (K) (radius = 50K) and 200K (radius = 100 K) base pairs centered at all loci of genetic markers available. The adjusted *p*-values are obtained by the adaptive Monte Carlo random permutation method and the lowest ones are calibrated to avoid the negative Monte Carlo estimation bias in multiple testing. The joint estimated significance cutoffs for the adjusted *p*-values related to the localization windows of all sizes are obtained empirically under the assumption of independent test statistics (Table S2).

The additional clinical measures were not available for all patients. In order to not lose the statistical power, only phenotypes available in all patients were selected for statistical analyses. These were: family history of AD; family history of mental disorders; average amount of alcohol consumed (absolute ethanol, grams per day) or TLFB (absolute ethanol, grams per day); STAI—State anxiety; STAI—Trait anxiety; OCDS; PACS; and VAS for alcohol craving. The statistical tests used for these additional analyses are listed in Table S1B,C. The Bonferroni-corrected GWS *p*-value in analyses listed in Table S1B was 7.289 × 10^−9^ (381,084 markers and 6 different phenotypes tested at α = 0.05 for three groups: the entire cohort, males, and females). In addition, we obtained Pearson’s correlations of clinical measures (Table S3) and ran statistical tests of their associations with the family history of AD (Table S4). Interestingly, the average amount of alcohol consumed per day (either self-reported or assessed with TFLB) did not correlate with the additional measures of anxiety or alcohol craving. Likewise, family history of AD was not associated with most clinical measures; only OCDS showed an association with family history of AD (*p*-value = 0.02).

Finally, results of previously published studies of psychiatric and addiction phenotypes, listed in the GWAS Catalog (https://www.ebi.ac.uk/gwas/home, accessed on 1 July 2021) [38], were intersected with our results, using as cutoff the replication *p*-value ≤ 5 × 10^−6^ [39].

### 2.5. Functional Annotation of Associated Loci

#### 2.5.1. Linkage Disequilibrium Blocks and Regulated Genes

Linkage disequilibrium (LD) blocks that contain associated SNPs were determined using methods described in [40]. Data from the Genotype-Tissue Expression (GTEx) Project were used to identify expression quantitative trait loci (eQTLs) in brain regions among associated variants. This Project was supported by the Common Fund of the Office of the Director of the National Institutes of Health, and by NCI, NHGRI, NHLBI, NIDA, NIMH, and NINDS. The data used for the analyses described in this manuscript were obtained from the GTEx Portal on 30 March 2022. Regulatory sequences, identified by the PsychENCODE Consortium (PEC) [41], namely, eQTLs, isoform percentage QTLs (isoQTLs), and enhancers, as well as top enhancers and promoters from the GeneCards Suite [42,43] were determined as described in [40]. In this paper, genes are identified by HUGO Gene Nomenclature Committee symbols (www.genenames.org, accessed on 1 July 2021) [44].

#### 2.5.2. Gene Networks

Known and predicted interactions of discovered genes were analyzed with String V.11.0b (https://version-11-0b.string-db.org/, accessed on 1 August 2021) [45]. For this analysis, full String network option and all active interaction sources except ‘gene fusion’ were used. As a minimum required interaction score the medium value of 0.4 was applied. Because the String database can work only with protein-coding genes, these genes were selected for the analysis. Among the 101 genes identified in the previous step, 80 were coding. Almost all discovered coding genes, except *RSPH6A* that has a high expression level in the liver according to one study [46], were confirmed to be brain-expressed by using Expression Atlas release 8 February 2022 (https://www.ebi.ac.uk/gxa/home, accessed on 1 June 2022). Biological pathways associated with the 80 coding genes were retrieved from the Kyoto Encyclopedia of Genes and Genomes (KEGG) database [47,48,49], release 101.0, by using the KEGG Mapper search tool. PEC network modules [50] were also checked for the presence of these coding genes, by retrieving information from the original publication (Table S8 of [50]) and from the Network Module Visualization web interface (https://pintolab.mssm.edu/papers/crossdisorder2018_netgraphs/, accessed on 1 July 2021). Finally, the Gene Ontology (GO) (http://geneontology.org, accessed on 1 July 2021) [51,52] top categories associated with the modules were also retrieved from the Network Modules Visualization web interface.

To extract previously published coding genes showing the strongest association with alcohol-related phenotypes, we used literature reviews published from 2019 describing results of GWASs of alcohol dependence and alcohol consumption [18,20,53,54]. The diagnoses in these studies were made using the *Alcohol Use Disorders Identification Test (AUDIT)*, *Diagnostic and Statistical Manual of Mental Disorders (DSM-IV)*, and *ICD-9/10*. We also extracted the best candidate genes from original reports, published from 2019, describing more recent (not included in the listed reviews) GWASs of AD (defined with *DSM-IV* and *ICD-9/10* criteria) [6], AD combined with problematic drinking (defined with *AUDIT-P*) [7], and alcohol consumption (including heavy consumption) [8,9,11]. Because we were comparing the published data with results obtained in a cohort with European Russian ancestry, we only included results from studies that used European-ancestry samples. In total, 120 genes from the previous studies were identified. Gene networks were visualized with Cytoscape 3.8.0 (https://cytoscape.org, accessed on 1 September 2022) [55]. The GO knowledgebase and the PANTHER classification system V.17.0 (http://pantherdb.org, accessed on 1 August 2021) [56,57] were used to evaluate enrichment for top biological processes associated with the interacting genes by applying Fisher’s exact test. The Benjamini–Hochberg False Discovery Rate (FDR) correction < 0.05 was also used, following recommendations [58]. The biological processes associated with the interacting genes were determined by matching against all Homo sapiens genes.

## 3. Results

### 3.1. Association Studies

Figure S1A–L shows Manhattan plots of the results of χ^2^ tests (the screening stage) for the main phenotype AD. The top *p*-values for the screening stage and results of additional appropriate tests are listed in Table S5A and Table 3. Dominant, codominant, and allelic tests of the screening stage (categorical data), as well as Yates-corrected χ^2^ tests indicated GWS associations with the marker rs220677 with *p*-values ranging from 1.33 × 10^−8^ to 2.11 × 10^−8^ (Table 3). These associations with AD were found only in females. Signal localization did not indicate GWS results (Table S6A–G lists the top hits of the adjusted *p*-values), but a signal in females closest to the GWS level (calibrated *p*-value = 8.9 × 10^−8^ under the dominant model) was discovered in the region on chr6:46821990-46916551 (Table S6F). This region includes the LD block (chr6:46867760-46879764, see Section 3.2) containing rs220677, significantly associated with AD in female patients. The genotype refinement of the region on chr6:46821990-46916551 using the 1000 Genomes data was not possible because of an insufficient imputation quality: 2383 of 3116 SNPs had the imputation metric dosage R-squared (DR2) below 0.1, which is less than the minimal recommended threshold of 0.3 [59].

Potential associations with additional phenotypes are listed in Table S5B,C. To investigate these, we retrospectively calculated the power of F-tests following a linear regression model given additive and equal effects of the alleles in the total population of 192 patients, as well as 26 female and 166 male patients (Figure S2A–C). The calculations of power were for a biallelic genetic marker with a realistic MAF = 0.1 (MAF = 0.3 in the female population), under HWE, for 381,084 × 6 tests (the number of markers after QC times the number of phenotypes tested) under α = 0.05. The calculations indicated that a population of 192 individuals is not large enough to allow discovering new associations. However, the obtained results may be used to replicate previously reported associations with relevant phenotypes.

In fact, there were several intersections between psychiatric and addiction phenotypes in the GWAS Catalog and our results (Table 3 and Table S5A,B). Alcohol craving assessed with PACS in female patients and brain region volumes (the region of interest indicated as ‘right vessel’) [60] shared associations with rs9842222. In addition, the same phenotype in female patients and neuroticism [61] were both associated with rs593531. Trait anxiety assessed with STAI also in female patients and proneness to anger [62] were both associated with rs2148710. Different results were obtained in the male and mixed (predominantly male) cohorts. AD in the mixed and male cohort and schizophrenia [63] shared associations with rs6868545, whereas alcohol craving assessed with PACS in the mixed cohort and schizophrenia [64] were both associated with rs9960767. Furthermore, alcohol craving assessed with PACS in the mixed cohort and multiple smoking behavior phenotypes together with general risk tolerance (https://www.ebi.ac.uk/gwas/variants/rs6265, accessed on 1 September 2022) [22] were associated with rs6265, whereas average amount of alcohol consumed per day in the mixed cohort and smoking initiation (a phenotype indicating whether an individual had ever smoked regularly) [22] were both associated with rs3810291.

Neither statistical test indicated an association with SNPs found in or near alcohol dehydrogenase and aldehyde dehydrogenase genes *ADH1A*, *ADH1B*, *ADH1C*, *ADH4*, *ADH5*, *ADH6*, *ADH7*, and *ALDH2* associated with alcohol phenotypes [7,9,10,14,17,20,21,22,23]. Among previously reported SNPs, the GSA includes only rs1229984 (*ADH1B*), rs1789891 (*ADH1B* and *ADH1C*), and rs671 (*ALDH2*). Rs1229984 had no genotype calls and the 1000 Genomes-based imputation of the entire *ADH1B* gene region was not successful (443 of 660 SNPs had the imputation metric dosage R-squared (DR2) below 0.1), which could indicate that an important association was missed. At the same time, it is unclear whether this marker could be truly useful given the sample size of the present study. Although rs1229984 is an extremely important variant associated with alcohol phenotypes [6,7,8,10,11,13,14,17,20,21,22,23], its MAF is low in populations with European ancestry and significant associations could be achieved only with large sample sizes [65]. The association study results for rs1789891 were carefully examined, but the lowest *p*-values were way above the significance threshold (nearly 3 × 10^−3^ for trait anxiety in males; data not shown). Finally, rs671 was excluded by QC thresholds because its MAF in the population under study was 0.00354, while the attempted 1000 Genomes-based imputation of the entire *ALDH2* gene region was not successful (1613 of 2074 SNPs had the imputation metric dosage R-squared (DR2) below 0.1).

### 3.2. Gene Networks

The novel associated and replicated SNPs are found within LD blocks, fragments of the genome transmitted through generations. These fragments contain several regulatory elements—eQTLs, isoQTLs, and enhancers—which regulate expression of genes in different tissues. The genes themselves might be found outside of an LD block that contains their regulatory elements (reviewed in Figure 3 of [40]). The LD blocks discovered in the present study, regulatory elements, 80 coding genes, and 21 RNA genes are listed in Table S7. The associated KEGG pathways that include at least two genes listed in Table S7 are listed in Table 4. The pathways include ‘Alcoholism’, ‘Dopaminergic synapse’, ‘Glutamatergic synapse’, and ‘Long-term potentiation’.

The SNP rs12527172 is in LD with rs220677 associated with AD in females. Rs12527172 (chr6:46869468) is an eQTL for the gene *SLC25A27* (ENSG00000153291) as reported by the PEC [41], and this SNP is also an eQTL in basal ganglia for the genes *ADGRF5* and *TDRD6* as reported by GTEx. The web resource of the PsychENCODE Project (http://resource.psychencode.org/, accessed on 1 July 2021) containing the file “DER-08a_hg38_eQTL.significant.txt” was used to retrieve the information about *SLC25A27*. This gene is listed among the best functional candidates (Table 3 and Table S7), because it is found in the PEC Network Module ‘geneM1′ with the top GO term ‘Synapse’, is functionally connected with *UCP2* and *UCP3* found in the discovered gene network (see below), has neuroprotective roles in the developing brain cortex, and is associated with schizophrenia (for more details, see Discussion). Although coding exons 10 to 12 of the gene *ADGRF5* are found in the candidate region chr6:46867760-46879764 and its expression is regulated by rs12527172, a careful analysis of its biological functions did not confirm its likely role in the pathogenesis of AD. This gene is not found in the discovered network, or in PEC Network Modules, or in relevant KEGG pathways (Table S7 and Table 4). There are no previously reported associations with addiction or psychiatric traits, except one report of an association of the rare missense variant rs149197213 in exon 6 of this gene with suicide [66]. Despite this, although we did not consider *ADGRF5* among the best functional candidate genes in the present study, further investigations are needed before the role of *ADGRF5* in the pathogenesis of AD can be completely ruled out.

**Figure 4:**
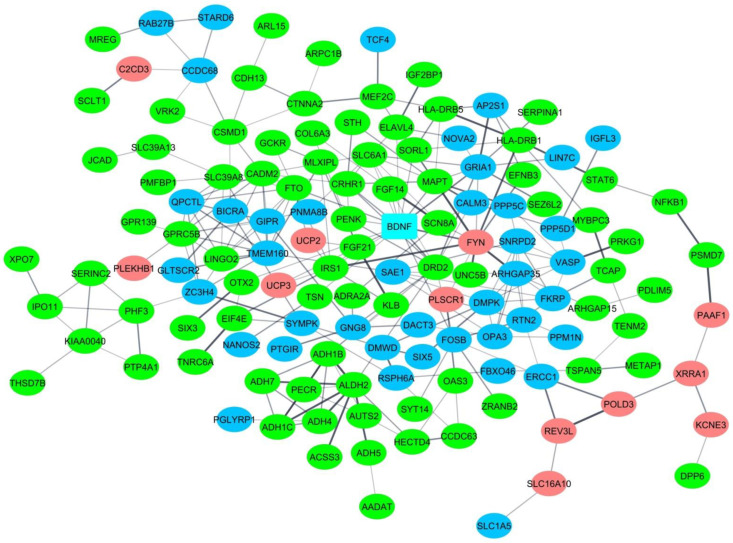
Interaction network of genes associated with AD and related phenotypes. The network is predicted by the String database. Genes colored in pink are associated with AD and related clinical measures in female patients; genes colored in blue are associated with AD and related clinical measures in male patients and the entire (predominantly male) cohort; genes colored in green were previously reported in the literature. The gene *BDNF* that was replicated in the present study and revealed in previous GWASs of alcohol phenotypes is colored in aquamarine. The thickness of edges indicates the strength of data support in terms of confidence scores: medium (0.4), high (0.7), highest (0.9). *BDNF* has the greatest number of connections (sixteen) with other nodes.

The 80 coding genes (Table S7), as well as 120 previously published coding genes (Table S8), were analyzed for interactions using the String database. Interestingly, 135 (67.8%) of the totaling 199 genes directly interact in a single network, without additional interactors added by the database (Figure 4). In particular, 54 genes (67.5%) among the 80 genes discovered in the present study participate in this network, whereas 82 (68.3%) out of the 120 previously published genes are also part of this network. String estimated that the probability of these gene interactions being due to chance alone is <10^−16^.

*BDNF* is found in the LD block chr11:27583087-27710436 that contains rs6265, associated with alcohol craving in the mixed cohort and a number of smoking behaviour phenotypes (Table S7). This important gene was both discovered in this study and in previous GWASs of alcohol consumption and problematic alcohol use [7,9] (Table S8). *BDNF* is the most prominent hub gene in the discovered network: it has the highest number of connections—sixteen—with other nodes (Figure 4).

The GO enrichment analysis of biological processes associated with the 135 interacting genes indicated ‘ethanol oxidation’ and ‘ethanol metabolic process’ that are driven by ethanol-metabolizing genes. This analysis also revealed several biological processes central in brain development, function, and plasticity, including: ‘cell morphogenesis involved in neuron differentiation’, ‘axon development’, ‘regulation of synapse structural plasticity’, ‘regulation of trans-synaptic signaling’, ’modulation of chemical synaptic transmission’, and ‘learning or memory’ (Table 5).

## 4. Discussion

AD is a common brain disorder with a number of abnormalities in opioid, dopaminergic, glutamatergic, and GABAergic neurotransmitter systems [1]. At least half of factors contributing to the etiology of AD are genetic [2,5], while genome-wide studies confirm a complex nature of this disorder, indicating not only polygenicity [6,7,8,9,10,11,12], but also pleiotropy [7,13,14,15,16,17].

The aim of the present investigation was to run the first GWAS in Russia in a cohort of patients with AD in whom additional clinical measures were also recorded (such as alcohol craving, amount of alcohol consumed per day, and anxiety symptoms). Different results were discovered in male and female patients, offering a confirmation of previous psychiatric genomics data [67]. Specifically, the investigation revealed a novel GWS association, supported by the signal localization approach, of AD with rs220677 in the female patients. In addition, associations with seven other SNPs listed in previous GWASs of psychiatric and addiction traits (brain region volumes, neuroticism, proneness to anger, schizophrenia, smoking behavior phenotypes, and general risk tolerance) were differently replicated in female and male patients (besides the main diagnosis, the associated phenotypes were alcohol craving, trait anxiety, and average amount of alcohol consumed per day). The eight associated loci contain several brain-specific regulatory elements that determine the expression of 80 protein-coding genes. A bioinformatic analysis of these and 120 other previously published coding genes associated with alcohol phenotypes revealed that 67.8% of them directly interact in a single network. *BDNF* that was reported in previous GWASs of alcohol consumption and problematic alcohol use [7,9] (Table S8) and replicated in the present GWAS (Table S7 and Table 3) is the most significant hub gene in the discovered network (Figure 4). It has sixteen connections with other nodes, which places it at the top of the list.

### 4.1. Sex Differences and AD

Males and females have significant differences in the structure and function of the brain [68]. The starting point leading to two distinct neurodevelopmental paths is the presence of the Y chromosome in males and expression of several genes on both X chromosomes that escape inactivation in females (besides the pseudoautosomal regions) [69]. Different transcriptomes are likely to be at the core of biological factors that determine differences in the brain structure and in the incidence and clinical picture of psychiatric disorders [70]. The transcriptomes are further modified by different sex hormone levels (also arising from the sex-specific expression of genes): the hormones bind to their respective nuclear receptors that act as transcription factors. These biological events define sex differences. Another significant factor that may determine male- or female-specific clinical presentation of psychiatric disorders is cultural norms that define gender differences. These may translate in ways boys and girls are brought up and in imposed roles of men and women in the society. These external influences also converge as biological factors, because they translate as changes in epigenetic profiles, and thus, in further changes in transcriptomes [71].

Social norms seem to be a factor that defines the significant differences in rates of male and female problematic drinking, even in countries where the alcohol consumption is widespread [72,73,74]. For example, in South Korea and Canada, the ratio of male to female problematic drinking is 5 and 6, respectively [73,75]. Despite the lower incidence of problematic drinking, women with AD more often present with comorbid anxiety and depressive disorders [74,75,76]. This could be explained by the higher prevalence of anxiety and depressive disorders in women [68,76,77]. AD is thus a clear example of a psychiatric disorder with a gender- and sex-specific presentation.

Our results support these data by revealing different genetic associations in male and female patients with AD. In female patients, there were genetic associations with AD, alcohol craving, and anxiety, supporting the previous reports of higher rates of comorbid AD and anxiety in women. Some of replicated genetic associations were previously reported for neuroticism and proneness to anger, which further suggests that AD in female patients genetically overlaps with anxiety and mood disorders. On the other hand, our results suggest that AD, alcohol craving, and average amount of alcohol consumed per day in male patients are genetically related to schizophrenia and smoking behavior phenotypes, possibly indicating a different genetic architecture of AD in men [13].

AD in the female cohort seems to have a stronger genetic component, as several genetic associations were found despite its quite small size. These results could be explained by a higher threshold in females in terms of the liability to develop AD [17], because social norms act as protective environmental factors. On the other hand, the higher heritability estimates could be due to a smaller size, i.e., an insufficient statistical power of female samples [17], and necessitate further investigations. Previous reports of SNP heritability indicated either higher [17] or lower [5] heritability of alcohol use disorders (as a proxy of AD) in female patients.

### 4.2. Function of the Discovered Genes

The 80 coding genes discovered in the present GWAS are active in pathways ‘Alcoholism’ (one of the key genes is *BDNF*), ‘Dopaminergic synapse’, ‘Glutamatergic synapse’, and ‘Long-term potentiation’ (Table 4). In addition, 40 of the 80 genes are found in PEC Network Modules with the top GO terms ‘Synapse’; ‘Synaptic vesicle cycle’; ‘Anterograde trans-synaptic signaling’ (this network module includes *BDNF*); ‘Neuron development, neuronal cell body, ion channel activity, axon guidance’; ‘Axon ensheathment’; ‘Signaling receptor activity, cell surface’; ‘Cilium organization, microtubule’; ‘Mitochondrial membrane, oxidative phosphorylation’; ‘Inflammatory response’; ‘Leukocyte activation, signaling receptor activity, cell surface’ (Table S7).

In addition, this study revealed a *BDNF*-centered gene network associated with alcohol dependence and other related phenotypes. Despite numerous sex differences, genes discovered in the female and male cohorts interact in the same network. The discovered interaction network contains several genes active in biological processes central to brain development, function, and plasticity, such as ‘cell morphogenesis involved in neuron differentiation’, ‘regulation of synapse structural plasticity’, and ‘regulation of trans-synaptic signaling’ (Table 5). The advantage of discovering gene networks is that they offer a possibility to develop new drug targets [78]. The best functional candidate genes discovered in the present GWAS and found in the network are described below (also see Table S7 where these genes are indicated in bold and Table 3).

By binding to its receptor tropomyosin receptor kinase B (TrkB), brain-derived neurotrophic factor, encoded by *BDNF,* regulates neuronal development and function and is important in synaptic plasticity [79,80]. Levels of this growth factor are altered in a number of psychiatric disorders and substance use phenotypes: for example, they are reduced in AD [81] and schizophrenia [82], but are increased in nicotine dependence [83]. *BDNF* is associated with problematic alcohol use [7], alcohol consumption [9], general risk tolerance and externalizing behavior [21], as well as various smoking behavior phenotypes (for example, [22]). Associations with more psychiatric and substance use phenotypes are listed at https://www.ebi.ac.uk/gwas/genes/BDNF, accessed on 1 September 2022. The functional variant rs6265, associated with alcohol craving in the present study (a replicated association), is a GTEx eQTL for multiple genes in frontal cortex, basal ganglia, anterior cingulate cortex, hippocampus, substantia nigra, and hypothalamus (Table 3) and it changes the amino acid Valine to Methionine (GTG → ATG). This variant has been extensively studied in psychiatry and neurology (https://www.ncbi.nlm.nih.gov/CBBresearch/Lu/Demo/LitVar/#!?query=rs6265, accessed on 1 September 2022). In particular, it is associated with a higher risk and earlier occurrence of relapse in AD [24] and with brain activation in precuneus, superior parietal lobule, and posterior cingulate in individuals with AD exposed to the taste of alcohol [25]. In addition, there is some evidence of association of this variant with AD in schizophrenic patients [26] and with resiliency in the context of problematic alcohol use [27].

*FYN*, associated with trait anxiety in female patients, codes for a non-receptor protein tyrosine kinase member of the Src family that plays an important role in numerous aspects of neurodevelopment and in regulation of glutamatergic neurotransmission [84]. In particular, FYN regulates the activity of N-methyl-D-aspartate (NMDA) and α-amino-3-hydroxy-5-methyl-4-isoxazolepropionic acid (AMPA) receptor subunits by phosphorylation [84] and its activation by heavy alcohol consumption seems to explain alcohol dependent behavioral phenotypes [85]. Candidate-gene association studies indicated that several variants in *FYN* are associated with AD [86,87]; furthermore, *FYN* is a part of a gene network associated with AD [19]. As shown by previous GWASs, this kinase is associated not only with proneness to anger, but also with schizophrenia [88].

One of the AMPA receptor subunit genes is *GRIA1*, associated with AD in the mixed cohort. Expression of this gene is increased by alcohol consumption [85] and a mouse model indicates that this subunit is important in synaptic plasticity and for inhibiting the reaction to irrelevant stimuli [89]. Furthermore, the gene has multiple associations with schizophrenia [63,90,91,92,93,94].

Another important factor in glutamatergic neurotransmission is calmodulin 3, encoded by *CALM3*, associated with the amount of alcohol consumed per day in the mixed cohort. The function of calmodulin is to bind Ca^2+^ that enters through the NMDA receptor pore; this binding activates calcium/calmodulin-dependent protein kinase II (CamKII) that in turn regulates trafficking of the AMPA receptor to the cell membrane [85]. These events define one of the forms of synaptic plasticity: long-term memory, believed to be affected in progression to AD [85].

*TCF4* associated with alcohol craving in the mixed cohort codes for a transcription factor that regulates expression of genes active during brain development, as well as in various aspects of neurotransmission [95,96]. Pathways regulated by this transcription factor are enriched in genes associated with neurodevelopmental disorders, including schizophrenia [95]. This gene is associated with a myriad of psychiatric phenotypes, including problematic alcohol use [7], schizophrenia [90], and neuroticism [97] (the full list of studies is found at https://www.ebi.ac.uk/gwas/genes/TCF4, accessed on 1 September 2021).

Finally, the expression of *SLC25A27* is regulated by a PEC eQTL (rs12527172) from the LD block that contains rs220677 and this gene is not predicted to make part of the discovered gene network. However, its function could be linked to the pathogenesis of AD. The gene codes for solute carrier family 25 member 27, a mitochondrial uncoupling protein (UCP). It is associated with AD in female patients, while *UCP2* and *UCP3* found in the predicted gene network (Figure 4) are associated with alcohol craving, also in female patients (Table 3 and Table S7). The three encoded proteins are responsible for the proton leak across the inner membrane of mitochondria, thus uncoupling oxidative phosphorylation from ATP synthesis. In this way, SLC25A27 and UCP2 are believed to protect neurons against reactive oxygen species [98]. In addition, SLC25A27 might play neuroprotective roles in the developing brain cortex [98], and its gene is associated with the treatment-resistant form of schizophrenia, a neurodevelopmental disorder [99]. Interestingly, as also indicated by the predicted gene network (Figure 4), UCPs are implicated in the same pathways than fibroblast growth factor 21 (FGF21) and its coreceptor β-Klotho (KLB) [100]. *FGF21* and *KLB* are furthermore associated with alcohol consumption [8,9,101] (Table S8). These pathways activate in response to consumption of sugar or ethanol, offering confirmation that nutrient and alcohol intake are in part regulated by the same genes [101].

### 4.3. Limitations

The study has several limitations. The main one is the small group of patients, particularly female patients. Despite this quantitative limitation, detailed phenotyping [102] and an increased ‘control to case’ ratio [103] deployed in this study should enable using a smaller sample of patients. The next limitation is that the new association of rs220677 with AD in female patients requires further validation in independent samples and the same is true for the replicated associations with psychiatric and addiction traits. The third limitation is that the biological mechanisms of the associated genetic variants and their potential role in the pathogenesis of AD need to be investigated using in vitro and in vivo laboratory models. The fourth limitation is that the study only analyzed data from subjects with European ancestry, which does not represent the wealth of genetic diversity of human populations. Including populations with other than ethnic Russian descent in future studies, for example, including cohorts from one of the many indigenous peoples of the Russian Federation [104,105], will fill this gap. Finally, we were comparing results from differently ascertained cohorts, which has proven to be a major concern in genetic studies [106].

## 5. Conclusions

This work offers additional confirmation of the previously published data by indicating that several genes behind the pathogenesis of AD are different in male and female patients, although implicated molecular mechanisms are functionally connected. In particular, the study reveals a central role of *BDNF* in the pathogenesis of AD. There is also additional confirmation of the genetic basis of sex-specific psychiatric comorbidities of alcohol dependence (e.g., anxiety symptoms in women). The discovered gene network may contain clues to the understanding of the pathogenesis and to the discovery of new treatment options of AD.

## Figures and Tables

**Table 1:** (A) Clinical characteristics of the entire cohort of individuals with AD. (B) Clinical characteristics of the male patients with AD. (C) Clinical characteristics of the female patients with AD.

Phenotype	Density (%)	Mean	Standard Deviation	Yes	No
(**A**)					
Alcohol Dependence (AD)	100			224	0
Family history of AD	100			117	107
Family history of mental disorders	100			10	214
Average amount of alcohol consumed, either self-reported or assessed with TFLB (absolute ethanol, grams per day)	100	101.3	82.1		
TLFB, Number of heavy drinking days	50	24.8	21.9		
TLFB, Number of drinking days	50	9	8.7		
TLFB, Number of sobriety days	50	56.1	24.7		
STAI—State anxiety	100	39.8	9.6		
STAI—Trait anxiety	100	42.6	9.5		
OCDS	100	12.1	12.2		
PACS	100	3.8	4.9		
VAS for alcohol craving	100	1.6	1.6		
HAS	50	7	5.7		
MADRS	50	5.9	5.4		
CGI—Severity	50	4.1	0.6		
Average number of cigarettes smoked daily	33.5	14.6	9.3		
(**B**)					
Alcohol Dependence (AD)	100			192	0
Family history of AD	100			104	88
Family history of mental disorders	100			7	185
Average amount of alcohol consumed, either self-reported or assessed with TFLB (absolute ethanol, grams per day)	100	107	85.8		
TLFB, Number of heavy drinking days	51.6	25.1	21.8		
TLFB, Number of drinking days	51.6	8.1	17		
TLFB, Number of sobriety days	51.6	56.7	24.1		
STAI—State anxiety	100	39.8	9.9		
STAI—Trait anxiety	100	42	9.4		
OCDS	100	11.6	12		
PACS	100	3.7	4.5		
VAS for alcohol craving	100	1.5	1.5		
HAS	51.6	7.2	5.8		
MADRS	51.6	6	5.4		
CGI—Severity	51.6	4.1	0.6		
Average number of cigarettes smoked daily	33.3	16.3	8.5		
(**C**)					
Alcohol Dependence (AD)	100			32	0
Family history of AD	100			13	19
Family history of mental disorders	100			3	29
Average amount of alcohol consumed, either self-reported or assessed with TFLB (absolute ethanol, grams per day)	100	67.15	40.84		
TLFB, Number of heavy drinking days	40.6	21.9	23.5		
TLFB, Number of drinking days	40.6	16.3	28.5		
TLFB, Number of sobriety days	40.6	51.8	29.6		
STAI—State anxiety	100	39.9	7.4		
STAI—Trait anxiety	100	46.4	9.1		
OCDS	100	14.7	13.2		
PACS	100	4.5	6.7		
VAS for alcohol craving	100	2	2.1		
HAS	40.6	6.1	4.3		
MADRS	40.6	4.5	4.7		
CGI—Severity	40.6	4.1	0.6		
Average number of cigarettes smoked daily	34.4	4.7	7.3		

Phenotypes are defined in the text. Density indicates the proportion of patients with available clinical information related to the listed phenotypes. Mean and standard deviation are indicated for quantitative measures. Yes/No indicates numbers of patients in each of the two corresponding categories.

**Table 2:** (A) The sequential QC procedure for cases and controls. (B) The sequential QC procedure for cases only.

Filtration Step	Variants Removed	People Removed	Variants Remaining	People Remaining
(**A**)				
SNP missingness (<0.2)	24,963	0	617,861	1283
Missingness per individual (<0.2)	0	7	617,861	1276
SNP missingness (<0.02)	75,494	0	542,367	1276
IND missingness (<0.02)	0	26	542,367	1250
Sex discrepancy	0	12	542,367	1238
Autosomes only	13,562	0	528,805	1238
MAF < 0.01	116,798	0	412,007	1238
hwe 1 × 10^−6^	43	0	411,964	1238
Heterozygocity outliers	0	0	411,964	1238
Inbreeding (autosomal het)	0	0	411,964	1238
Relatedness (IBD)	0	29	411,964	1209
MDS outlier	0	1	411,964	1208
SNPs removed by Illumina	374	0	411,590	1208
SNPs removed from dbSNP	4	0	411,586	1208
Final	231,238	75	411,586	1208
(**B**)				
Selection of samples	0	1059	642,824	224
SNP missingness (<0.2)	26,960	0	615,864	224
Missingness per individual (<0.2)	0	7	615,864	217
SNP missingness (<0.02)	93,387	0	522,477	217
IND missingness (<0.02)	0	6	522,477	211
Sex discrepancy	0	6	522,477	205
Autosomes only	12,785	0	509,692	205
MAF < 0.01	128,251	0	381,441	205
hwe 1 × 10^−10^	9	0	381,432	205
Heterozygocity outliers	0	0	381,432	205
Inbreeding (autosomal het)	0	0	381,432	205
Relatedness (IBD)	0	12	381,432	193
MDS outlier	0	1	381,432	192
SNPs removed by Illumina	345	0	381,087	192
SNPs removed from dbSNP	3	0	381,084	192
Final	261,740	1091	381,084	192

**Table 3:** GWAS top results.

Test Name	Phenotype	Sex	Chr	Position (hg38)	SNP	*p*-Value (Screening)	*p*-Value (Yates-Corrected χ^2^)	*p*-Value (Fisher’s Exact)	Replication	*p*-Value (Original Results)	GTEx eQTL ^b^	Top Candidate Gene
ALC:INC/SPB:F/CAT/D	Alcohol dependence	Female	6	46,872,709	**rs220677 ^a^**	**1.33 × 10^−8^**	**1.33 × 10^−8^**	1.47 × 10^−7^	N/A	N/A	No	*SLC25A27*
ALC:INC/SPB:F/CAT/CD						**1.85 × 10^−8^**	**1.85 × 10^−8^**	1.47 × 10^−7^				
ALC:INC/SPB:F/CAT/A						**2.11 × 10^−8^**	**2.11 × 10^−8^**	6.38 × 10^−7^				
ALC:INC/SPB:F/GLM:PC1/D						1.65 × 10^−7^	**1.33 × 10^−8^**	1.47 × 10^−7^				
ALC:INC/SPB:F/GLM:PC1/CD						1.07 × 10^−6^	**1.85 × 10^−8^**	1.47 × 10^−7^				
ALC:INC/SPB:F/GLM:PC1/A						1.05 × 10^−6^	**2.11 × 10^−8^**	6.38 × 10^−7^				
ALC:INC/SPB/CAT/CD		Mixed	5	153,115,323	rs6868545	1.49 × 10^−7^	1.49 × 10^−7^	8.25 × 10^−6^	Schizophrenia	7 × 10^−7^	No	*GRIA1*
ALC:INC/SPB:M/CAT/CD		Male				9.62 × 10^−7^	9.62 × 10^−7^	3.04 × 10^−5^				
**Test Name**	**Phenotype**	**Sex**	**Chr**	**Position (hg38)**	**SNP**	***p*-Value (Screening)**	***p*-Value (Linear Model)**	***p*** **-Value (GLM Quasi-Poisson)**	**Replication**	***p*-Value (Original Results)**	**GTEx eQTL**	**Top Candidate Gene**
ALC:TAI/SPB:F/GLM/A	STAI—Trait anxiety	Female	6	111,801,023	rs2148710	4.34 × 10^−6^	3.97 × 10^−5^	4.34 × 10^−6^	Anger (proneness to anger)	3 × 10^−8^	Yes, in frontal cortex, basal ganglia, and hippocampus ^c^	*FYN*
ALC:PACS/SPB:F/GLM/CD	Penn alcohol craving scale		3	146,650,400	rs9842222	5.93 × 10^−7^	2.73 × 10^−4^	5.93 × 10^−7^	Brain region volumes (region: right vessel)	5 × 10^−9^	No	
ALC:PACS/SPB:F/GLM/D						1.03 × 10^−6^	5.88 × 10^−5^	1.03 × 10^−6^				
ALC:PACS/SPB:F/GLM/A			11	74,406,417	rs593531	2.89 × 10^−6^	4.94 × 10^−6^	2.89 × 10^−6^	Neuroticism	2 × 10^−6^	Yes, in brain cortex, basal ganglia, hippocampus, and hypothalamus	*UCP2/3*
ALC:PACS/SPB/LM/R		Mixed	11	27,658,369	rs6265	5.27 × 10^−7^	5.27 × 10^−7^	1.20 × 10^−4^	Smoking behavior phenotypes; General risk tolerance	9 × 10^−29 d^	Yes, in frontal cortex, basal ganglia, anterior cingulate cortex, hippocampus, substantia nigra, and hypothalamus	*BDNF*
ALC:PACS/SPB/LM/CD						1.03 × 10^−6^	1.03 × 10^−6^	1.16 × 10^−4^				
ALC:PACS/SPB/LM/R			18	55,487,771	rs9960767	1.22 × 10^−6^	1.22 × 10^−6^	1.03 × 10^−3^	Schizophrenia	4 × 10^−9^	No	*TCF4*
ALC:PACS/SPB/LM/CD						3.68 × 10^−6^	3.68 × 10^−6^	2.45 × 10^−3^				
ALC:ATF/SPB/GLM:VERS/D	Average amount of alcohol consumed		19	47,065,746	rs3810291	1.55 × 10^−7^	5.06 × 10^−6^	1.55 × 10^−7^	Smoking initiation (ever smoked regularly)	2 × 10^−8^	Yes, in brain cortex, basal ganglia, and hypothalamus	*CALM3*
ALC:ATF/SPB/GLM:VERS/CD						1.09 × 10^−6^	3.01 × 10^−5^	1.09 × 10^−6^				
ALC:ATF/SPB:M/GLM:VERS/D		Male				1.49 × 10^−6^	3.19 × 10^−5^	1.49 × 10^−6^				

^a^ GWS results are indicated in bold. ^b^ This indicates whether the associated SNP is a GTEx expression quantitative trait locus. ^c^ Only tissues sampled from the central nervous system, except the spinal cord and the cerebellum, are indicated. ^d^ The *p*-values reported in the GWAS catalog range from 9 × 10^−29^ to 7 × 10^−7^.

**Table 4:** KEGG Mapper search results for the 80 coding genes discovered in this study.

Pathways	Genes
**hsa05034 Alcoholism—Homo sapiens (human) (4)**	**hsa:2354 FOSB; FosB proto-oncogene, AP-1 transcription factor subunit**
	**hsa:627 BDNF; brain derived neurotrophic factor**
	**hsa:808 CALM3; calmodulin 3**
	**hsa:94235 GNG8; G protein subunit gamma 8**
hsa04024 cAMP signaling pathway—Homo sapiens (human) (4)	hsa:2696 GIPR; gastric inhibitory polypeptide receptor
	hsa:2890 GRIA1; glutamate ionotropic receptor AMPA type subunit 1
	hsa:627 BDNF; brain derived neurotrophic factor
	hsa:808 CALM3; calmodulin 3
hsa04611 Platelet activation—Homo sapiens (human) (4)	hsa:2534 FYN; FYN proto-oncogene, Src family tyrosine kinase
	hsa:2909 ARHGAP35; Rho GTPase activating protein 35
	hsa:5739 PTGIR; prostaglandin I2 receptor
	hsa:7408 VASP; vasodilator stimulated phosphoprotein
hsa05016 Huntington disease—Homo sapiens (human) (4)	hsa:1175 AP2S1; adaptor related protein complex 2 subunit sigma 1
	hsa:27113 BBC3; BCL2 binding component 3
	hsa:2890 GRIA1; glutamate ionotropic receptor AMPA type subunit 1
	hsa:627 BDNF; brain derived neurotrophic factor
**hsa04728 Dopaminergic synapse—Homo sapiens (human) (3)**	**hsa:2890 GRIA1; glutamate ionotropic receptor AMPA type subunit 1**
	**hsa:808 CALM3; calmodulin 3**
	**hsa:94235 GNG8; G protein subunit gamma 8**
hsa05031 Amphetamine addiction—Homo sapiens (human) (3)	hsa:2354 FOSB; FosB proto-oncogene, AP-1 transcription factor subunit
	hsa:2890 GRIA1; glutamate ionotropic receptor AMPA type subunit 1
	hsa:808 CALM3; calmodulin 3
hsa05200 Pathways in cancer—Homo sapiens (human) (3)	hsa:27113 BBC3; BCL2 binding component 3
	hsa:808 CALM3; calmodulin 3
	hsa:94235 GNG8; G protein subunit gamma 8
hsa04015 Rap1 signaling pathway—Homo sapiens (human) (3)	hsa:25865 PRKD2; protein kinase D2
	hsa:7408 VASP; vasodilator stimulated phosphoprotein
	hsa:808 CALM3; calmodulin 3
hsa05022 Pathways of neurodegeneration—multiple diseases—Homo sapiens (human) (3)	hsa:2890 GRIA1; glutamate ionotropic receptor AMPA type subunit 1
	hsa:627 BDNF; brain derived neurotrophic factor
	hsa:808 CALM3; calmodulin 3
hsa04974 Protein digestion and absorption—Homo sapiens (human) (3)	hsa:10008 KCNE3; potassium voltage-gated channel subfamily E regulatory subunit 3
	hsa:117247 SLC16A10; solute carrier family 16 member 10
	hsa:6510 SLC1A5; solute carrier family 1 member 5
hsa04080 Neuroactive ligand-receptor interaction—Homo sapiens (human) (3)	hsa:2696 GIPR; gastric inhibitory polypeptide receptor
	hsa:2890 GRIA1; glutamate ionotropic receptor AMPA type subunit 1
	hsa:5739 PTGIR; prostaglandin I2 receptor
hsa04713 Circadian entrainment—Homo sapiens (human) (3)	hsa:2890 GRIA1; glutamate ionotropic receptor AMPA type subunit 1
	hsa:808 CALM3; calmodulin 3
	hsa:94235 GNG8; G protein subunit gamma 8
hsa04510 Focal adhesion—Homo sapiens (human) (3)	hsa:2534 FYN; FYN proto-oncogene, Src family tyrosine kinase
	hsa:2909 ARHGAP35; Rho GTPase activating protein 35
	hsa:7408 VASP; vasodilator stimulated phosphoprotein
hsa04014 Ras signaling pathway—Homo sapiens (human) (3)	hsa:627 BDNF; brain derived neurotrophic factor
	hsa:808 CALM3; calmodulin 3
	hsa:94235 GNG8; G protein subunit gamma 8
hsa01524 Platinum drug resistance—Homo sapiens (human) (3)	hsa:2067 ERCC1; ERCC excision repair 1, endonuclease non-catalytic subunit
	hsa:27113 BBC3; BCL2 binding component 3
	hsa:5980 REV3L; REV3 like, DNA directed polymerase zeta catalytic subunit
**hsa04724 Glutamatergic synapse—Homo sapiens (human) (2)**	**hsa:2890 GRIA1; glutamate ionotropic receptor AMPA type subunit 1**
	**hsa:94235 GNG8; G protein subunit gamma 8**
**hsa04720 Long-term potentiation—Homo sapiens (human) (2)**	**hsa:2890 GRIA1; glutamate ionotropic receptor AMPA type subunit 1**
	**hsa:808 CALM3; calmodulin 3**
hsa04723 Retrograde endocannabinoid signaling—Homo sapiens (human) (2)	hsa:2890 GRIA1; glutamate ionotropic receptor AMPA type subunit 1
	hsa:94235 GNG8; G protein subunit gamma 8
hsa05030 Cocaine addiction—Homo sapiens (human) (2)	hsa:2354 FOSB; FosB proto-oncogene, AP-1 transcription factor subunit
	hsa:627 BDNF; brain derived neurotrophic factor
hsa04151 PI3K-Akt signaling pathway—Homo sapiens (human) (2)	hsa:627 BDNF; brain derived neurotrophic factor
	hsa:94235 GNG8; G protein subunit gamma 8
hsa04722 Neurotrophin signaling pathway—Homo sapiens (human) (2)	hsa:627 BDNF; brain derived neurotrophic factor
	hsa:808 CALM3; calmodulin 3
hsa04010 MAPK signaling pathway—Homo sapiens (human) (2)	hsa:5536 PPP5C; protein phosphatase 5 catalytic subunit
	hsa:627 BDNF; brain derived neurotrophic factor
hsa01100 Metabolic pathways—Homo sapiens (human) (2)	hsa:283209 PGM2L1; phosphoglucomutase 2 like 1
	hsa:79147 FKRP; fukutin related protein
hsa04022 cGMP-PKG signaling pathway—Homo sapiens (human) (2)	hsa:7408 VASP; vasodilator stimulated phosphoprotein
	hsa:808 CALM3; calmodulin 3
hsa04270 Vascular smooth muscle contraction—Homo sapiens (human) (2)	hsa:5739 PTGIR; prostaglandin I2 receptor
	hsa:808 CALM3; calmodulin 3
hsa04380 Osteoclast differentiation—Homo sapiens (human) (2)	hsa:2354 FOSB; FosB proto-oncogene, AP-1 transcription factor subunit
	hsa:2534 FYN; FYN proto-oncogene, Src family tyrosine kinase
hsa04925 Aldosterone synthesis and secretion—Homo sapiens (human) (2)	hsa:25865 PRKD2; protein kinase D2
	hsa:808 CALM3; calmodulin 3
hsa05163 Human cytomegalovirus infection—Homo sapiens (human) (2)	hsa:808 CALM3; calmodulin 3
	hsa:94235 GNG8; G protein subunit gamma 8
hsa04670 Leukocyte transendothelial migration—Homo sapiens (human) (2)	hsa:2909 ARHGAP35; Rho GTPase activating protein 35
	hsa:7408 VASP; vasodilator stimulated phosphoprotein
hsa04371 Apelin signaling pathway—Homo sapiens (human) (2)	hsa:808 CALM3; calmodulin 3
	hsa:94235 GNG8; G protein subunit gamma 8
hsa05167 Kaposi sarcoma-associated herpesvirus infection—Homo sapiens (human) (2)	hsa:808 CALM3; calmodulin 3
	hsa:94235 GNG8; G protein subunit gamma 8
hsa05170 Human immunodeficiency virus 1 infection—Homo sapiens (human) (2)	hsa:808 CALM3; calmodulin 3
	hsa:94235 GNG8; G protein subunit gamma 8
hsa03460 Fanconi anemia pathway—Homo sapiens (human) (2)	hsa:2067 ERCC1; ERCC excision repair 1, endonuclease non-catalytic subunit
	hsa:5980 REV3L; REV3 like, DNA directed polymerase zeta catalytic subunit
hsa04530 Tight junction—Homo sapiens (human) (2)	hsa:7408 VASP; vasodilator stimulated phosphoprotein
	hsa:8189 SYMPK; symplekin scaffold protein
hsa04725 Cholinergic synapse—Homo sapiens (human) (2)	hsa:2534 FYN; FYN proto-oncogene, Src family tyrosine kinase
	hsa:94235 GNG8; G protein subunit gamma 8
hsa03420 Nucleotide excision repair—Homo sapiens (human) (2)	hsa:10714 POLD3; DNA polymerase delta 3, accessory subunit
	hsa:2067 ERCC1; ERCC excision repair 1, endonuclease non-catalytic subunit

Several pathways relevant to AD are indicated in bold.

**Table 5:** Gene Ontology enrichment analysis of biological processes, associated with the 135 genes in the network.

GO Biological Process Complete	Fold Enrichment	Raw *p*-Value	FDR
ethanol oxidation (GO:0006069)	84.11	2.16 × 10^−8^	1.69 × 10^−4^
ethanol metabolic process (GO:0006067)	45.42	1.48 × 10^−8^	2.32 × 10^−4^
regulation of biological quality (GO:0065008)	2.08	7.79× 10^−8^	4.07 × 10^−4^
response to inorganic substance (GO:0010035)	4.71	4.14 × 10^−7^	1.62 × 10^−3^
cell morphogenesis involved in neuron differentiation (GO:0048667)	5.01	1.14 × 10^−6^	3.58 × 10^−3^
response to oxygen-containing compound (GO:1901700)	2.71	2.11 × 10^−6^	5.52 × 10^−3^
primary alcohol metabolic process (GO:0034308)	11.16	4.93 × 10^−6^	1.10 × 10^−2^
neuron development (GO:0048666)	3.34	9.12 × 10^−6^	1.79 × 10^−2^
plasma membrane bounded cell projection morphogenesis (GO:0120039)	4.21	1.71 × 10^−5^	2.06 × 10^−2^
cell projection morphogenesis (GO:0048858)	4.18	1.86 × 10^−5^	2.08 × 10^−2^
response to lead ion (GO:0010288)	30.28	1.70 × 10^−5^	2.22 × 10^−2^
cell morphogenesis involved in differentiation (GO:0000904)	3.96	1.56 × 10^−5^	2.23 × 10^−2^
axon development (GO:0061564)	4.69	1.30 × 10^−5^	2.27 × 10^−2^
diterpenoid metabolic process (GO:0016101)	10.21	3.85 × 10^−5^	2.41 × 10^−2^
cell development (GO:0048468)	2.39	4.05 × 10^−5^	2.44 × 10^−2^
neuron projection development (GO:0031175)	3.47	3.43 × 10^−5^	2.45 × 10^−2^
neuron projection morphogenesis (GO:0048812)	4.25	1.56 × 10^−5^	2.45 × 10^−2^
response to metal ion (GO:0010038)	4.7	2.97 × 10^−5^	2.45 × 10^−2^
regulation of synapse structural plasticity (GO:0051823)	56.77	4.39 × 10^−5^	2.46 × 10^−2^
cellular component morphogenesis (GO:0032989)	3.65	3.78 × 10^−5^	2.47 × 10^−2^
startle response (GO:0001964)	23.29	4.26 × 10^−5^	2.47 × 10^−2^
plasma membrane bounded cell projection organization (GO:0120036)	2.8	3.32 × 10^−5^	2.48 × 10^−2^
fatty acid omega-oxidation (GO:0010430)	64.88	3.21 × 10^−5^	2.51 × 10^−2^
regulation of trans-synaptic signaling (GO:0099177)	4.32	2.92 × 10^−5^	2.54 × 10^−2^
regulation of hormone levels (GO:0010817)	3.9	3.76 × 10^−5^	2.56 × 10^−2^
modulation of chemical synaptic transmission (GO:0050804)	4.33	2.85 × 10^−5^	2.63 × 10^−2^
response to morphine (GO:0043278)	21.63	5.53 × 10^−5^	2.71 × 10^−2^
cell part morphogenesis (GO:0032990)	4.02	2.78 × 10^−5^	2.72 × 10^−2^
regulation of cell communication (GO:0010646)	1.88	5.08 × 10^−5^	2.75 × 10^−2^
regulation of signaling (GO:0023051)	1.87	5.27 × 10^−5^	2.75 × 10^−2^
carbohydrate homeostasis (GO:0033500)	6.18	6.16 × 10^−5^	2.76 × 10^−2^
response to alkaloid (GO:0043279)	9.27	6.40 × 10^−5^	2.79 × 10^−2^
response to isoquinoline alkaloid (GO:0014072)	21.63	5.53 × 10^−5^	2.80 × 10^−2^
axonogenesis (GO:0007409)	4.76	2.69 × 10^−5^	2.81× 10^−2^
cell projection organization (GO:0030030)	2.68	6.10 × 10^−5^	2.81× 10^−2^
glucose homeostasis (GO:0042593)	6.21	5.95 × 10^−5^	2.83 × 10^−2^
regulation of transport (GO:0051049)	2.28	6.89 × 10^−5^	2.92 × 10^−2^
terpenoid metabolic process (GO:0006721)	9.08	7.12 × 10^−5^	2.94 × 10^−2^
learning or memory (GO:0007611)	5.12	8.63 × 10^−5^	3.47 × 10^−2^
regulation of synapse organization (GO:0050807)	5.85	8.89× 10^−5^	3.49 × 10−2
regulation of transmembrane transport (GO:0034762)	3.53	9.91× 10^−5^	3.79 × 10^−2^
negative regulation of calcium ion transmembrane transporter activity (GO:1901020)	17.81	1.10× 10^−4^	4.01 × 10^−2^
regulation of synapse structure or activity (GO:0050803)	5.69	1.08 × 10^−4^	4.02 × 10^−2^
negative regulation of insulin secretion (GO:0046676)	17.3	1.22 × 10^−4^	4.35 × 10^−2^
response to external stimulus (GO:0009605)	2.02	1.27 × 10^−4^	4.43 × 10^−2^
behavior (GO:0007610)	3.43	1.30 × 10^−4^	4.45 × 10^−2^
neuron differentiation (GO:0030182)	2.69	1.42 × 10^−4^	4.73 × 10^−2^

## Data Availability

Custom code or new software was not developed in this study. All data needed to evaluate the conclusions in the paper are present in the paper and the Supplementary Materials. Additional data related to this paper (e.g., summary statistics) may be requested from the corresponding author Anastasia Levchenko.

## Supplementary Materials

The following supporting information can be downloaded at: https://www.mdpi.com/article/10.3390/biomedicines10123007/s1, Table S1A: List of SNP-based statistical tests of association and patient counts for AD (411586 SNPs); Table S1B: List of SNP-based statistical tests of association and patient counts for AD-related phenotypes (381084 SNPs); Table S1C: List of SNP-based statistical tests of association and patient counts for family history (381084 SNPs); Table S2: Correction for multiple localization windows usage obtained by simulations; Table S3: Pearson’s correlations of clinical measures; Table S4: Tests of associations with the family history of AD; Table S5A: GWAS results for AD (*p*-values < 5 × 10^−6^ and replicated associations); Table S5B: GWAS results for AD-related phenotypes (*p*-values < 5 × 10^−6^ and replicated associations); Table S5C: GWAS results for family history (*p*-values < 5 × 10^−6^ and replicated associations); Table S6A: Signal localization: top adjusted *p*-values (<10^−5^) and joint regions (localization radius 10 SNPs); Table S6B: Signal localization: top adjusted *p*-values (<10^−5^) and joint regions (localization radius 20 SNPs); Table S6C: Signal localization: top adjusted *p*-values (<10^−5^) and joint regions (localization radius 35 SNPs); Table S6D: Signal localization: top adjusted *p*-values (<10^−5^) and joint regions (localization radius 50 SNPs); Table S6E: Signal localization: top adjusted *p*-values (<10^−5^) and joint regions (localization radius 100 SNPs); Table S6F: Signal localization: top adjusted *p*-values (<10^−5^) and joint regions (localization radius 50,000 bp); Table S6G: Signal localization: top adjusted *p*-values (<10^−5^) and joint regions (localization radius 100,000 bp); Table S7: Genomic regions and genes associated with AD and related phenotypes; Table S8: Previously published genes associated with alcohol dependence or consumption; Figure S1A–L: Manhattan plots of the results of χ^2^ tests (screening stage) for the main phenotype AD; Figure S2A–C: Post-hoc estimations of statistical power for 381,084 × 6 GWAS tests under α = 0.05 (the linear model).
