# Supplementary material for: A Genome-Wide Association Study Reveals a BDNF-Centered Molecular Network Associated with Alcohol Dependence and Related Clinical Measures
Source: Biomedicines. 2022 Nov 22;10(12):3007. doi: 10.3390/biomedicines10123007 (PMC9775455; doi:10.3390/biomedicines10123007)
Supplement: Supplementary file 1 [file biomedicines-10-03007-s001.zip › Supplementary Figure Legends.docx]

# Supplementary Figure Legends

**Figure S1.** Manhattan plots of the results of χ^2^ tests (screening stage) for the main phenotype AD. (**A**) Mixed, allelic test; (**B**) mixed, codominant test; (**C**) mixed, dominant test; (**D**) mixed, recessive test; (**E**) males, allelic test; (**F**) males, codominant test; (**G**) males, dominant test; (**H**) males, recessive test; (**I**) females, allelic test; (**J**) females, codominant test; (**K**) females, dominant test; (**L**) females, recessive test.

**Figure S2.** Post-hoc estimations of statistical power for 381084×6 GWAS tests under α=0.05 (the linear model). (**A**) Estimations the mixed group (size = 192, MAF=0.1); (**B**) the group of female patients (size = 26, MAF = 0.3); and (**C**) the group of male patients (size = 166, MAF = 0.1).
