# Supplementary figures and images for: A Genome-Wide Association Study Reveals a BDNF-Centered Molecular Network Associated with Alcohol Dependence and Related Clinical Measures
Source: Biomedicines. 2022 Nov 22;10(12):3007. doi: 10.3390/biomedicines10123007 (PMC9775455; doi:10.3390/biomedicines10123007)

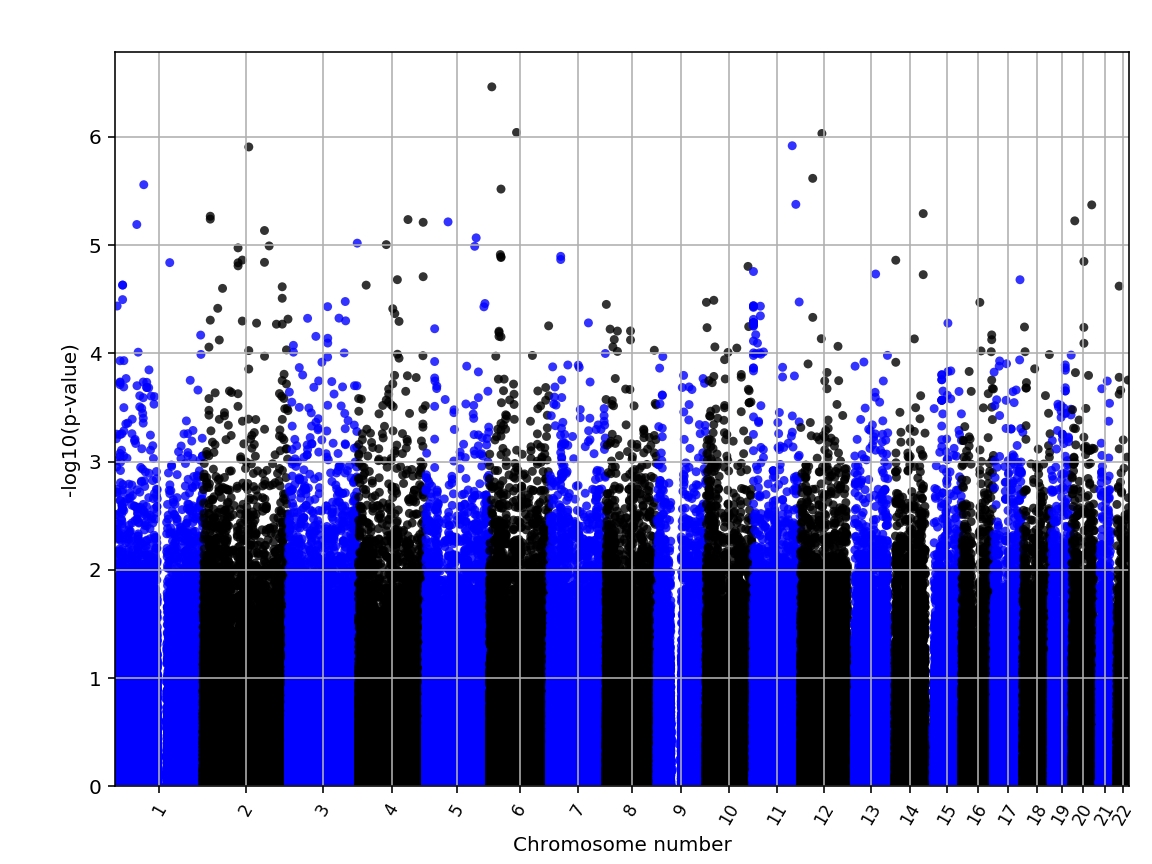

Supplement: Supplementary file 1 [file biomedicines-10-03007-s001.zip › Figure S1A.jpg]

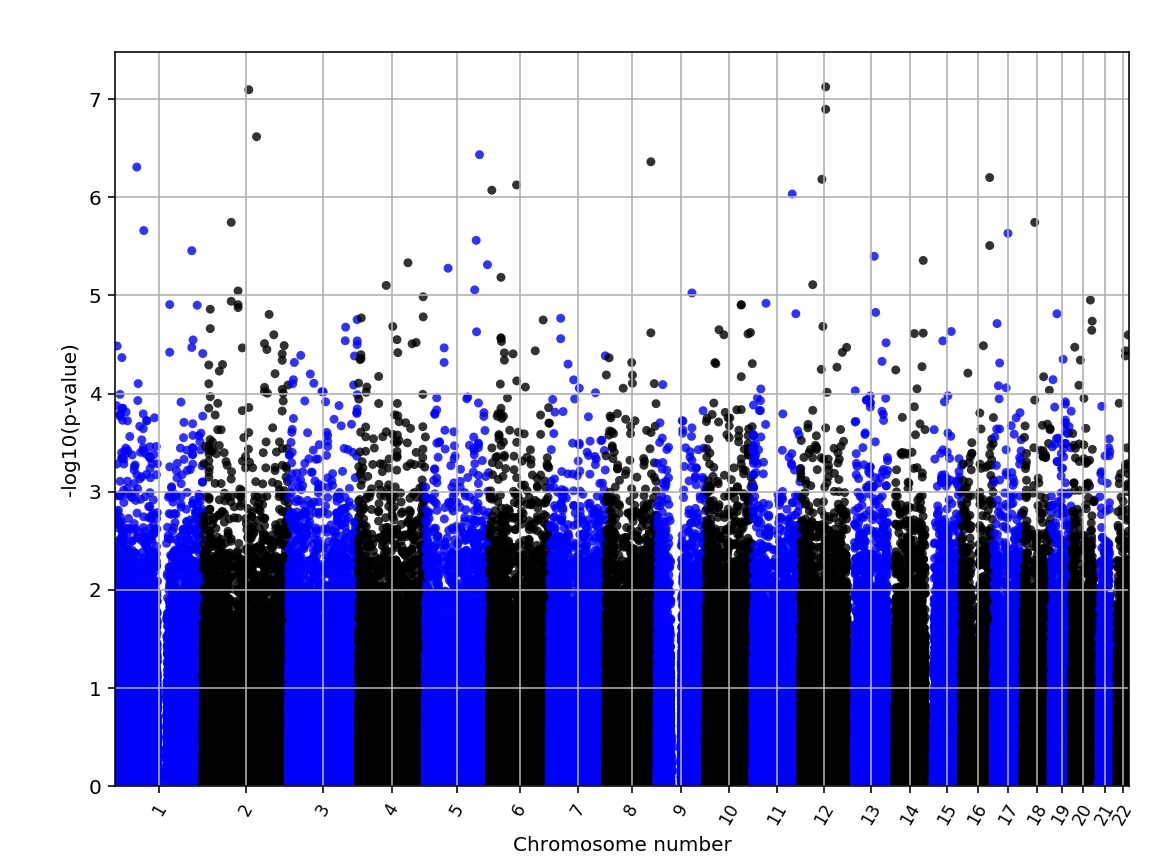

Supplement: Supplementary file 1 [file biomedicines-10-03007-s001.zip › Figure S1B.jpg]

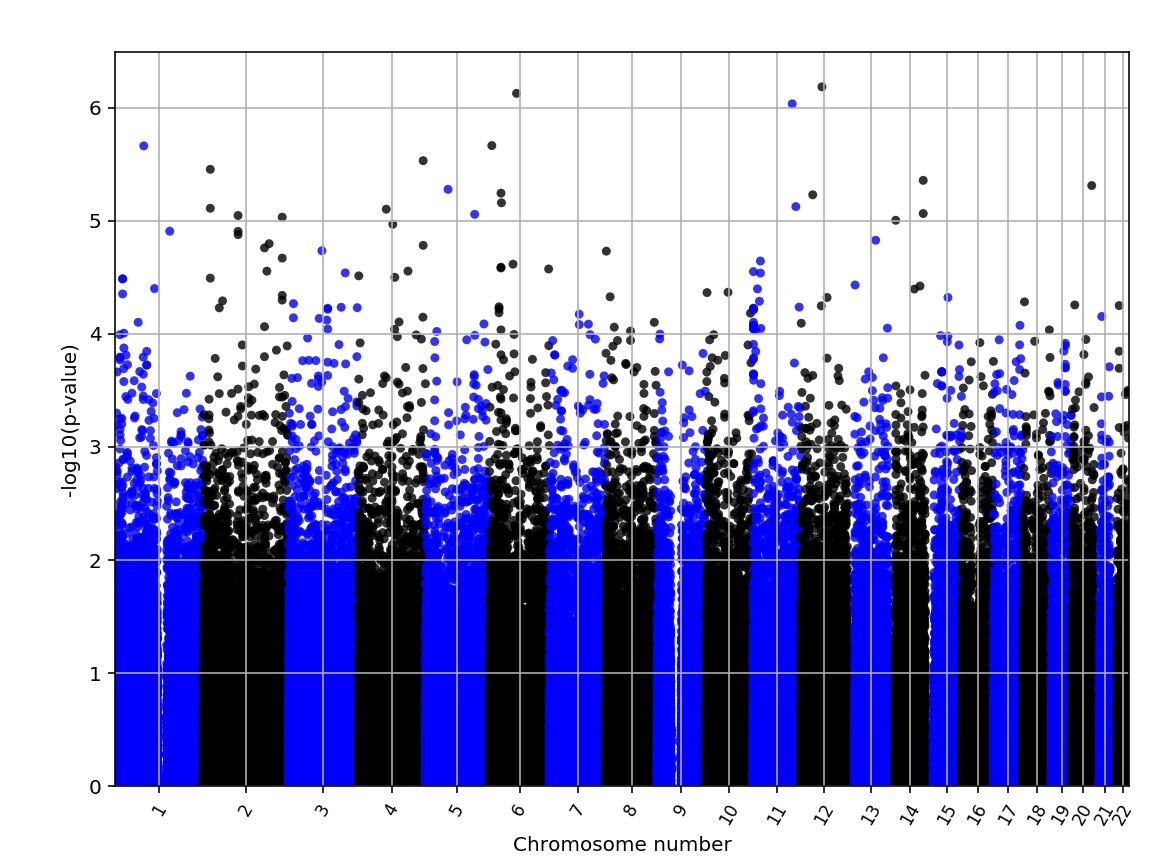

Supplement: Supplementary file 1 [file biomedicines-10-03007-s001.zip › Figure S1C.jpg]

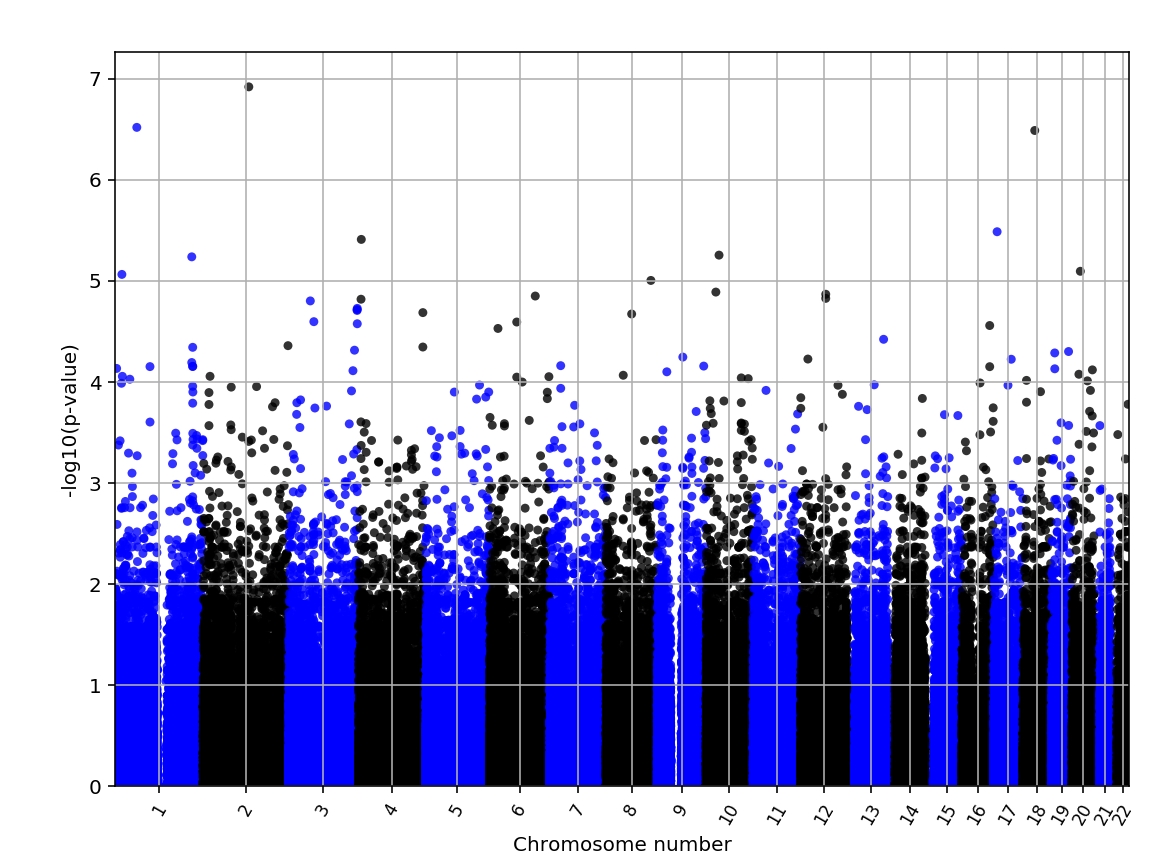

Supplement: Supplementary file 1 [file biomedicines-10-03007-s001.zip › Figure S1D.jpg]

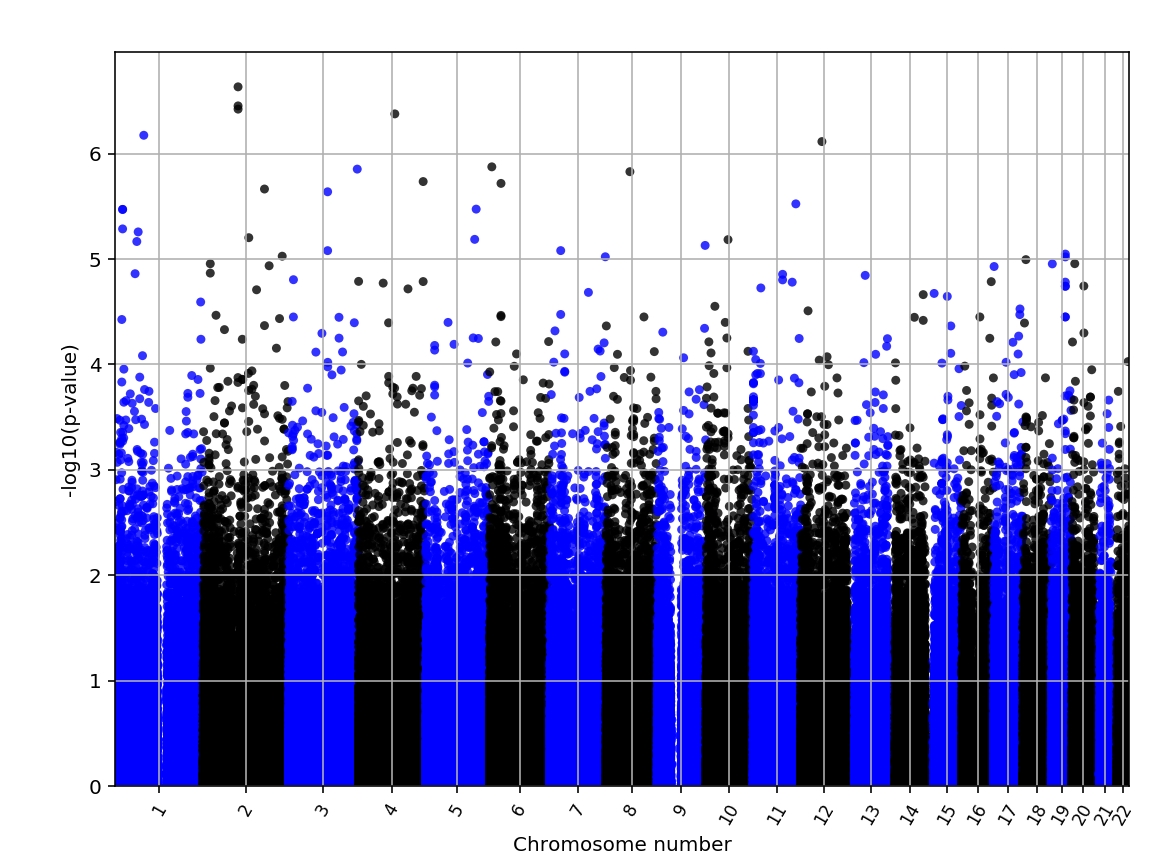

Supplement: Supplementary file 1 [file biomedicines-10-03007-s001.zip › Figure S1E.jpg]

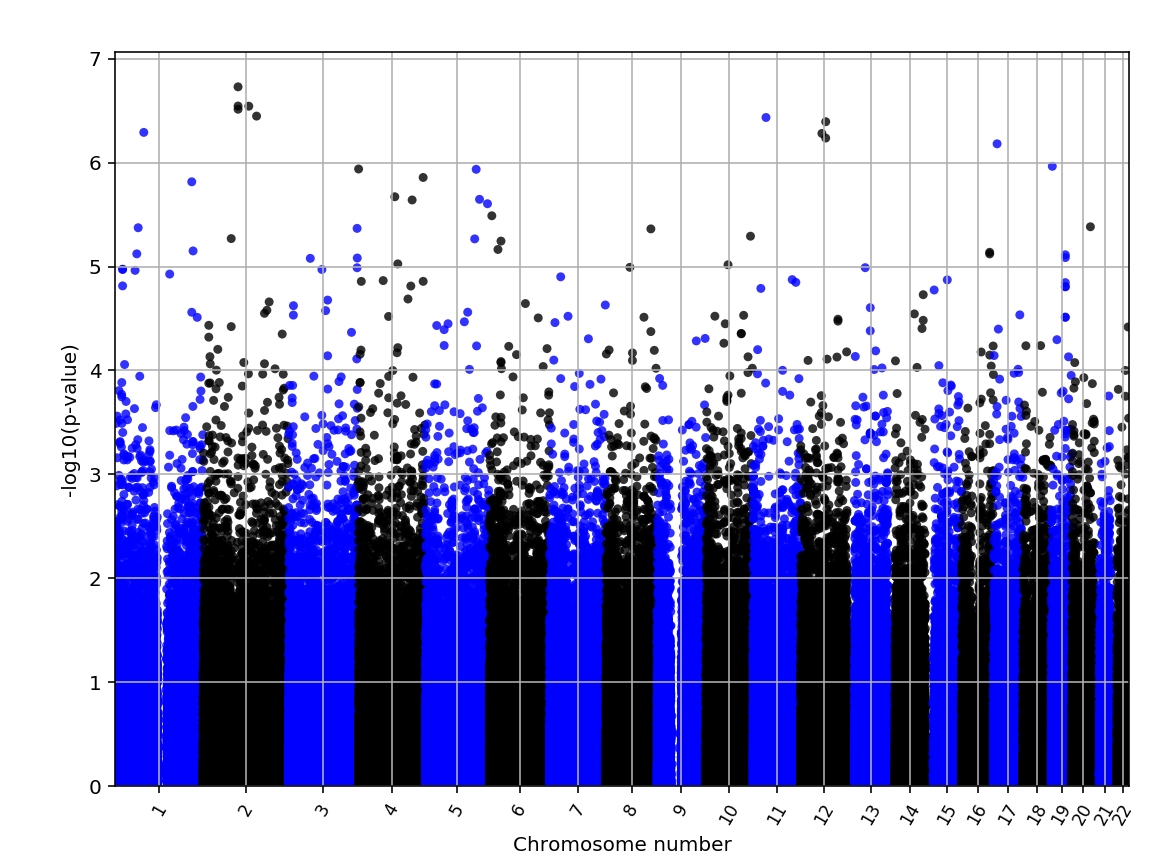

Supplement: Supplementary file 1 [file biomedicines-10-03007-s001.zip › Figure S1F.jpg]

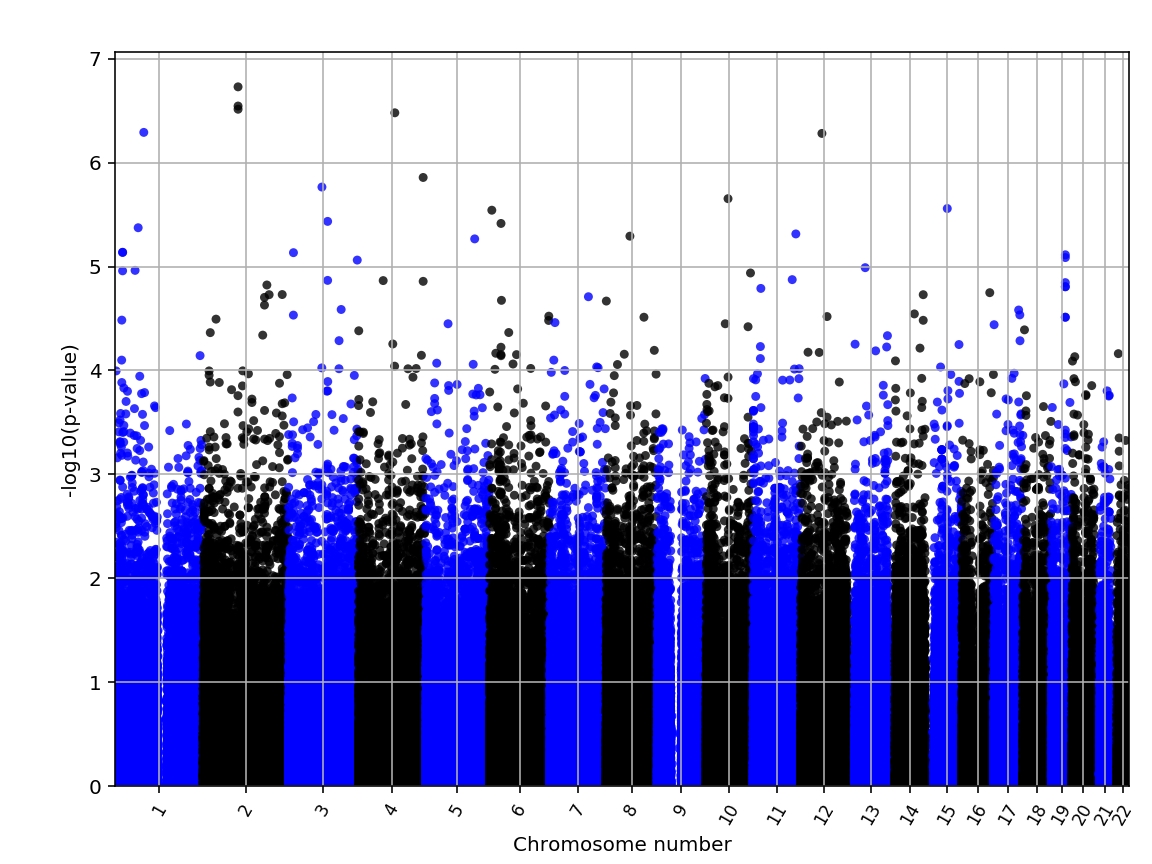

Supplement: Supplementary file 1 [file biomedicines-10-03007-s001.zip › Figure S1G.jpg]

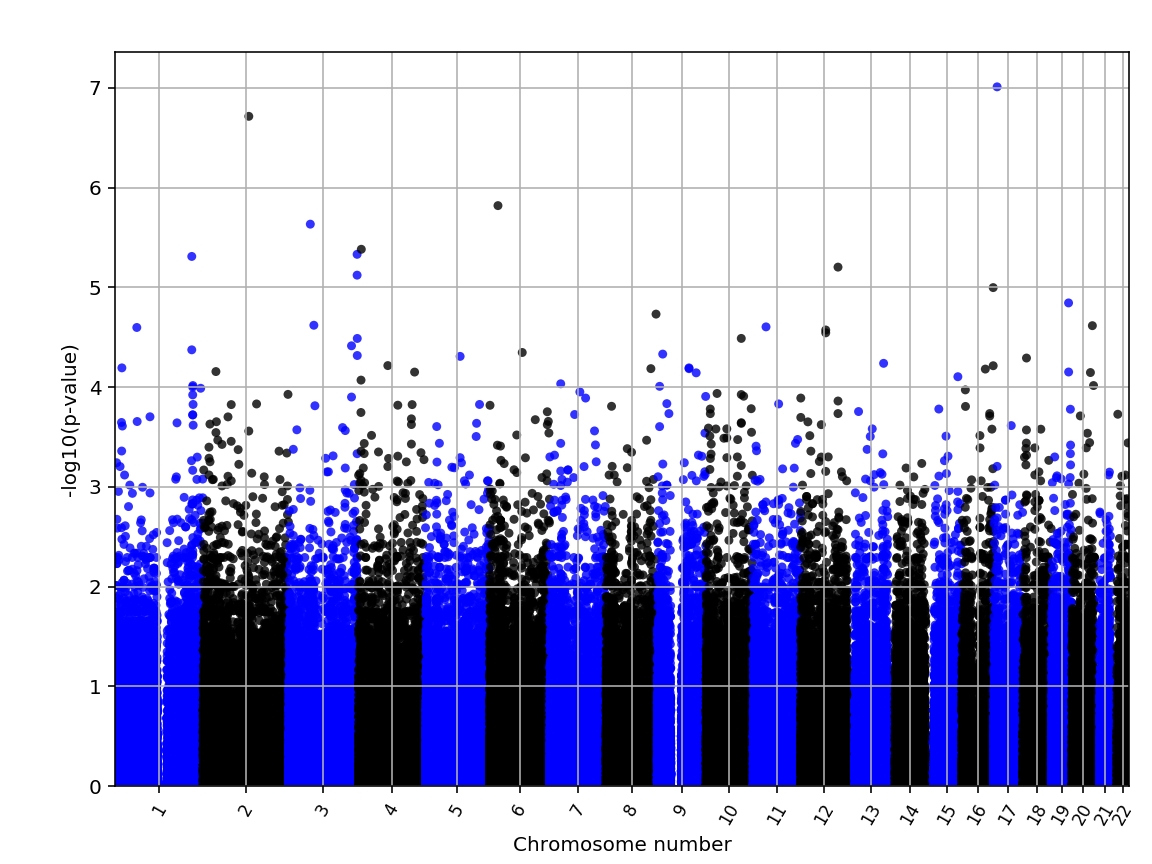

Supplement: Supplementary file 1 [file biomedicines-10-03007-s001.zip › Figure S1H.jpg]

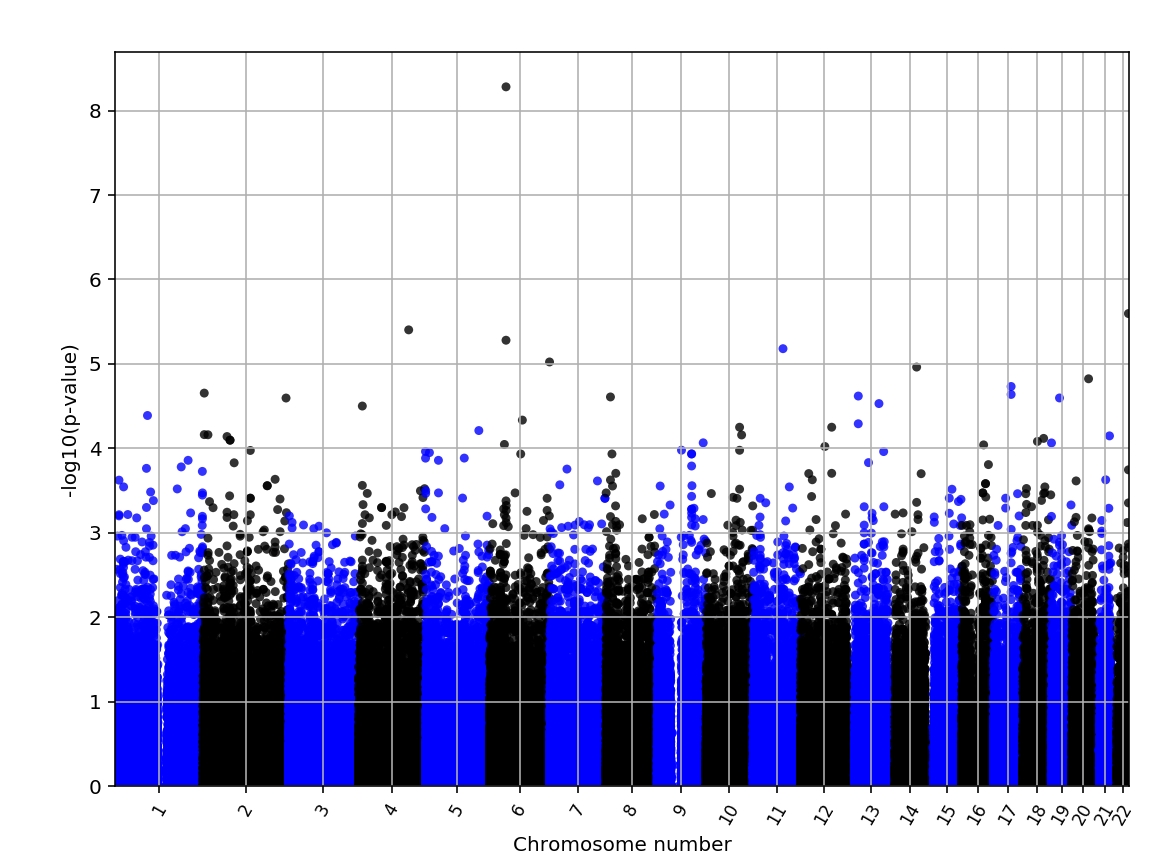

Supplement: Supplementary file 1 [file biomedicines-10-03007-s001.zip › Figure S1I.jpg]

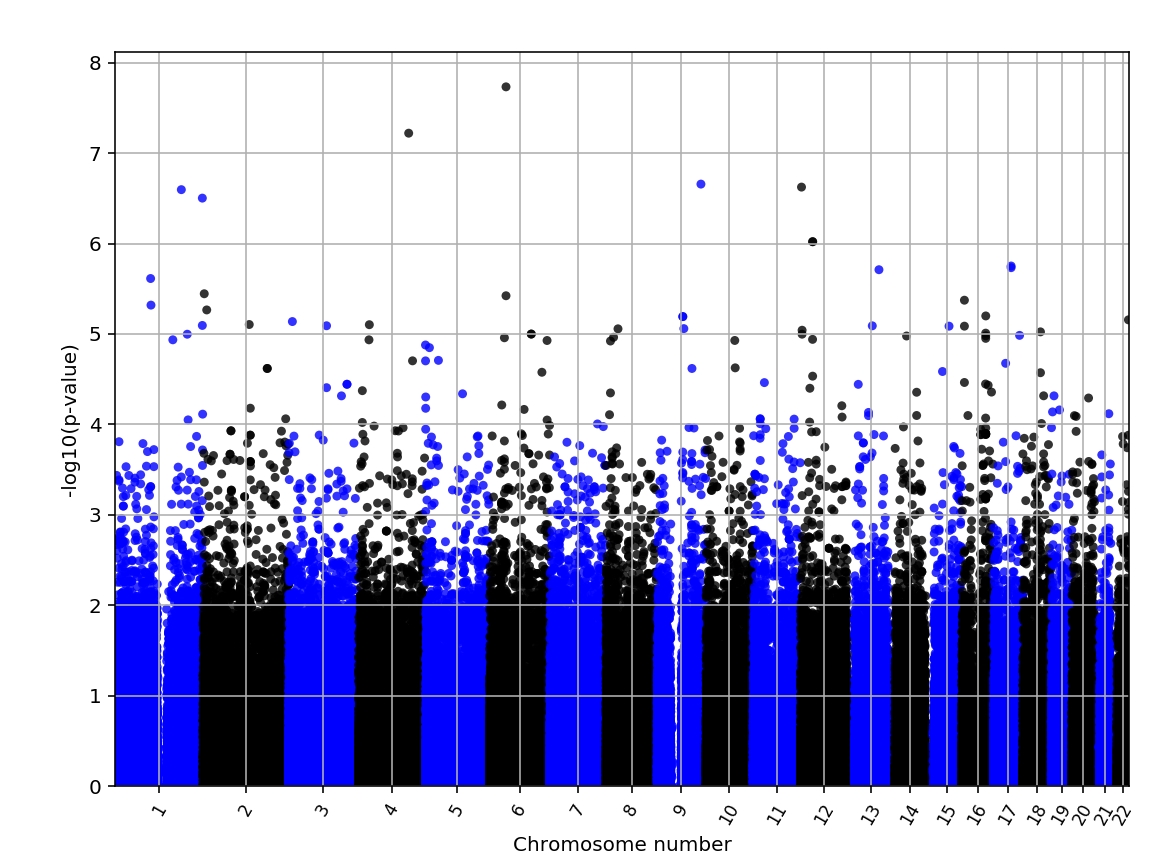

Supplement: Supplementary file 1 [file biomedicines-10-03007-s001.zip › Figure S1J.jpg]

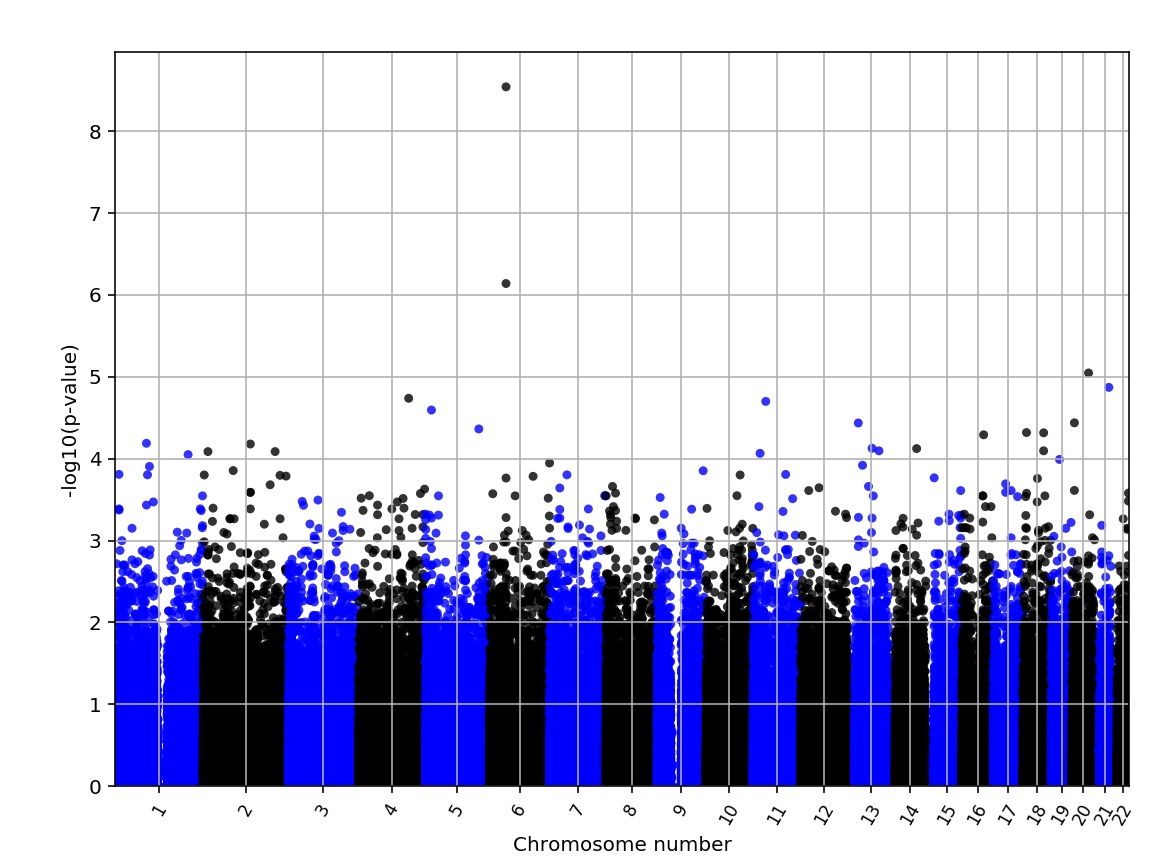

Supplement: Supplementary file 1 [file biomedicines-10-03007-s001.zip › Figure S1K.jpg]

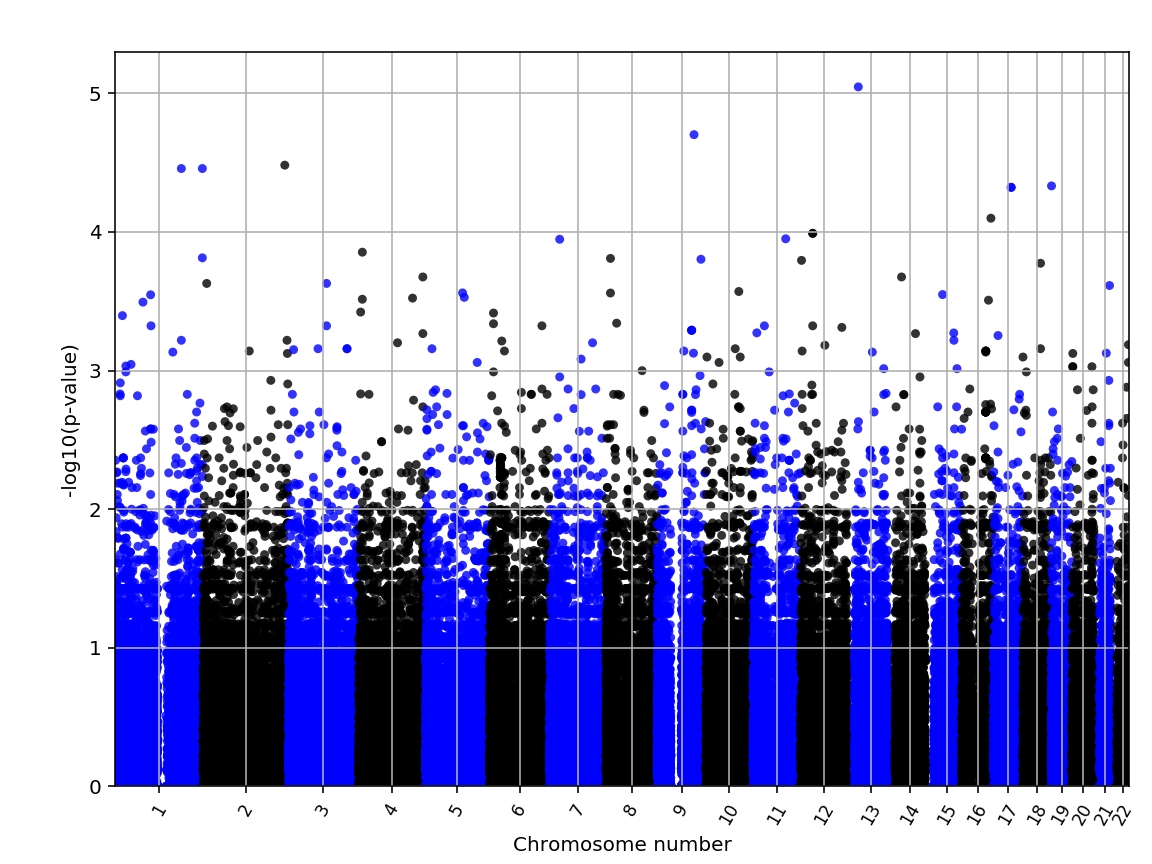

Supplement: Supplementary file 1 [file biomedicines-10-03007-s001.zip › Figure S1L.jpg]

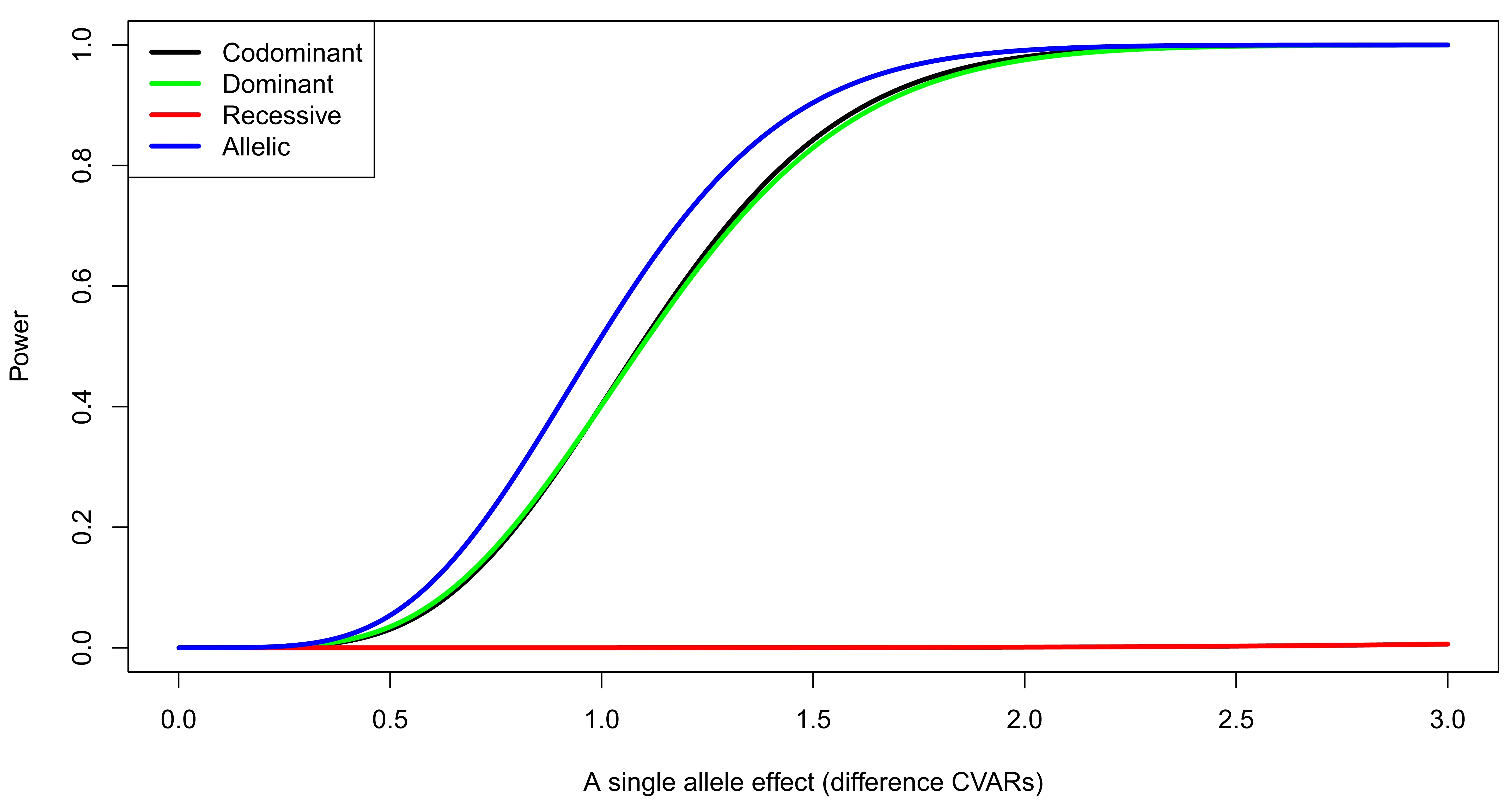

Supplement: Supplementary file 1 [file biomedicines-10-03007-s001.zip › Figure S2A.jpg]

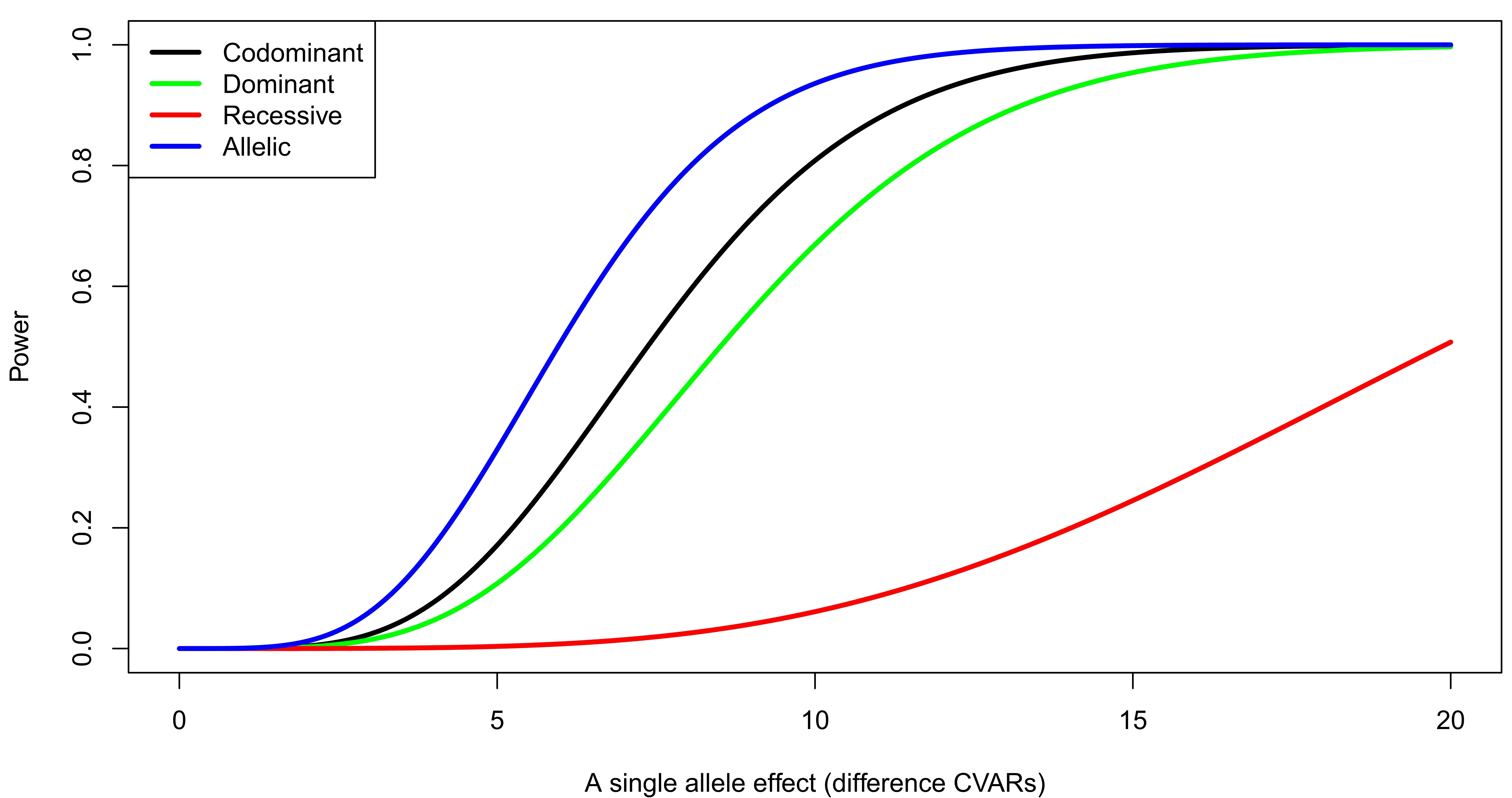

Supplement: Supplementary file 1 [file biomedicines-10-03007-s001.zip › Figure S2B.jpg]

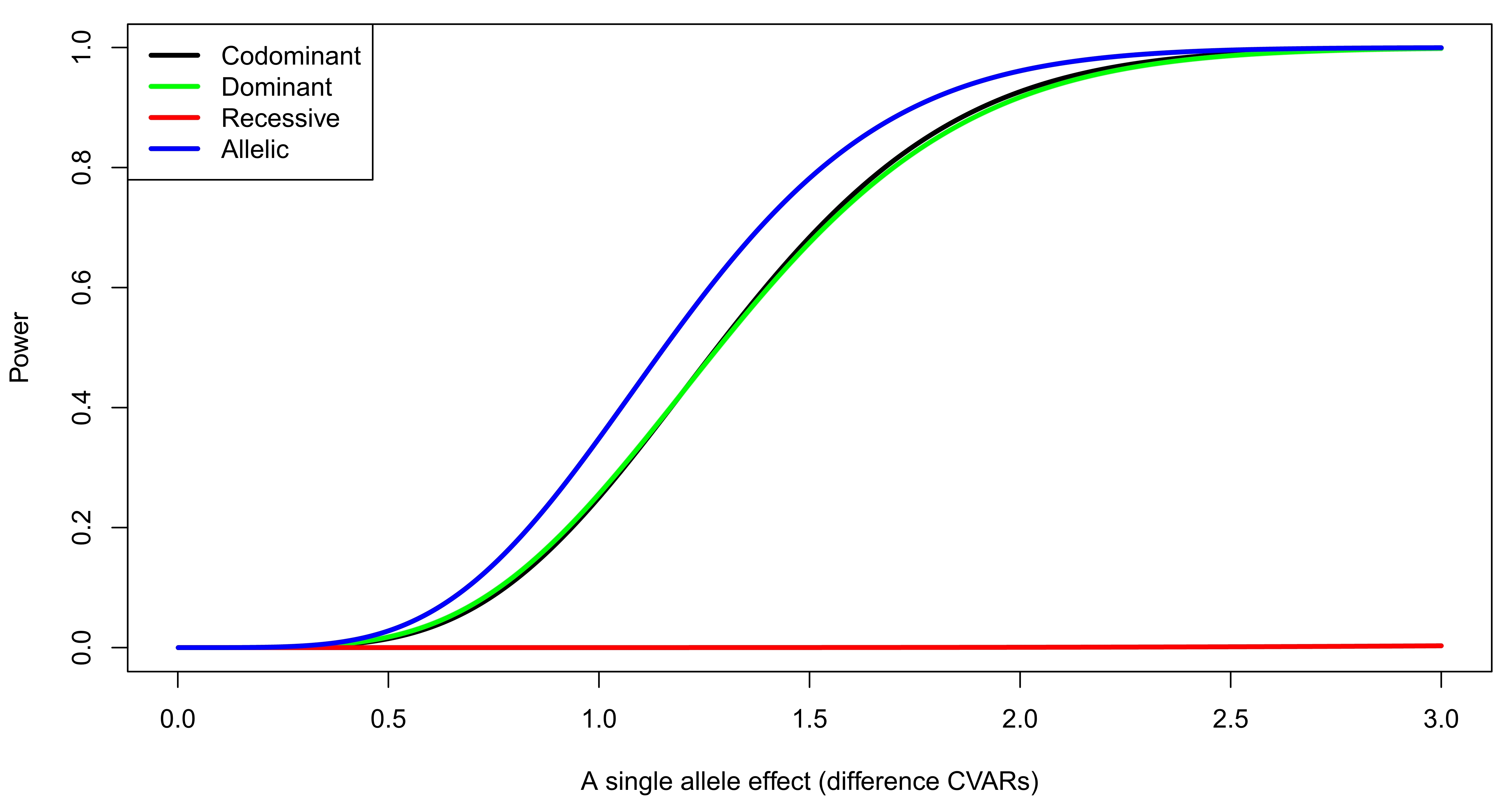

Supplement: Supplementary file 1 [file biomedicines-10-03007-s001.zip › Figure S2C.jpg]
